# A Genome-Wide Association Study of Nephrolithiasis in the Japanese Population Identifies Novel Susceptible Loci at 5q35.3, 7p14.3, and 13q14.1

**DOI:** 10.1371/journal.pgen.1002541

**Published:** 2012-03-01

**Authors:** Yuji Urabe, Chizu Tanikawa, Atsushi Takahashi, Yukinori Okada, Takashi Morizono, Tatsuhiko Tsunoda, Naoyuki Kamatani, Kenjiro Kohri, Kazuaki Chayama, Michiaki Kubo, Yusuke Nakamura, Koichi Matsuda

**Affiliations:** 1 Laboratory of Molecular Medicine, Human Genome Center, Institute of Medical Science, University of Tokyo, Tokyo, Japan; 2 Departments of Medical and Molecular Science, Division of Frontier Medical Science, Programs for Biomedical Research, Graduate School of Biomedical Sciences, Hiroshima University, Hiroshima, Japan; 3 Center for Genomic Medicine, The Institute of Physical and Chemical Research (RIKEN), Kanagawa, Japan; 4 Department of Nephro-Urology, Nagoya City University Graduate School of Medical Science, Nagoya, Japan; Georgia Institute of Technology, United States of America

## Abstract

Nephrolithiasis is a common nephrologic disorder with complex etiology. To identify the genetic factor(s) for nephrolithiasis, we conducted a three-stage genome-wide association study (GWAS) using a total of 5,892 nephrolithiasis cases and 17,809 controls of Japanese origin. Here we found three novel loci for nephrolithiasis: *RGS14-SLC34A1-PFN3-F12* on 5q35.3 (rs11746443; *P* = 8.51×10^−12^, odds ratio (OR) = 1.19), *INMT-FAM188B-AQP1* on 7p14.3 (rs1000597; *P* = 2.16×10^−14^, OR = 1.22), and *DGKH* on 13q14.1 (rs4142110; *P* = 4.62×10^−9^, OR = 1.14). Subsequent analyses in 21,842 Japanese subjects revealed the association of SNP rs11746443 with the reduction of estimated glomerular filtration rate (eGFR) (*P* = 6.54×10^−8^), suggesting a crucial role for this variation in renal function. Our findings elucidated the significance of genetic variations for the pathogenesis of nephrolithiasis.

## Author Summary

Although nephrolithiasis is one of the most common nephro-urological disorders with high prevalence (4%–9%) and extremely high recurrence rate (60% within ten years), little is known about the role of common variations in its pathogenesis. Through a GWAS using a total of 5,892 cases and 17,809 controls, we identified three novel nephrolithiasis loci: rs11746443, rs1000597, and rs4142110 (*P*<1×10^−8^). The top two significant SNPs, rs11746443 and rs1000597, are located upstream of the *SLC34A1* and the *AQP1* genes that play important roles in kidney function and the urine-concentration process, respectively. We also found that SNP rs11746443 is associated with the reduction of estimated glomerular filtration rate (eGFR), indicating the role of this variation in kidney function. Although nephrolithiasis is considered as one of the lifestyle-related diseases, the results of dietary intervention studies to reduce the recurrence incidence have been unsuccessful. Our findings could contribute to a better understanding of the pathogenesis of nephrolithiasis and lead to the development of new therapeutics.

## Introduction

Nephrolithiasis, also called kidney stone, is a common disorder which causes severe acute back pain and sometimes leads to severe complications such as pyelonephritis or acute renal failure. Lifetime prevalence of nephrolithiasis is estimated to be 4–9% in Japan [1], and nearly 60% of patients reveal recurrence within 10 years after their initial treatment [2]. Most of kidney stones are composed of calcium oxalate or calcium phosphate crystals [3]. Hypercalciuria, urinary tract infection, and alkaline urine are considered to cause urinary supersaturation and subsequently induce the formation of calcium stone. Westernized diet, obesity, and dehydration were also indicated their association with nephrolithiasis [4], [5]. In addition, a family history was reported to increase the disease risk (2.57 times higher) in males [6], and the concordance rate of the disease in monozygotic twins was significantly higher than that in dizygotic twins (32.4% vs. 17.3%) [7], indicating a pivotal role of genetic factors in its etiology. In 2009, a genome wide association study (GWAS) in Caucasian revealed a significant association of the *CLDN14* gene with nephrolithiasis and bone mineral density [8]. To investigate the genetic factors that are associated with the risk of nephrolithiasis in Japanese population, we conducted three-stage GWAS (Figure S1 and Table S1).

## Results/Discussion

In GWAS, we genotyped 1,000 Japanese patients with nephrolithiasis and 7,936 non-nephrolithiasis controls using Human Omni Express BeadChip. Patients with struvite, cystine, ammonium acid urate, and uric acid stone, or secondary nephrolithiasis caused by drugs, hyperparathyroidism, or kidney deformity were excluded from our analysis, since their underlying pathology is different from that of calcium nephrolithiasis. After standard quality-control filtering (call rate ≥0.99 in cases and controls, Hardy-Weinberg *P*≥1×10^−6^ in controls), we conducted principal component analysis and found that all subjects were of Asian ancestry (Figure S2). We evaluated the association of SNPs with nephrolithiasis by Cochran-Armitage trend test. As a result, the genomic inflation factor was calculated to be 1.123 (Figure S3). To adjust population stratification, we used two methods, logistic regression analysis using top principle components as covariate and the association analysis using 904 cases and 7,471 controls belonging to the Hondo cluster, the major cluster of Japanese population [9]. As a result, the genomic inflation factors for these analyses were improved to be 1.054 (adjustment by PC1 and PC2) and 1.042 (Hondo cluster), respectively (Figure S4a, S4b). Finally, we decide to use only Hondo cluster samples in GWAS to minimize likelihood of false positive associations. Although no SNP achieved the GWAS significance threshold (*P*<5×10^−8^), we selected top 100 SNPs (*P*≤7.85×10^−5^) showing the smallest *P*-value for further association analysis (Figure S4c and Table S2).

We selected 64 SNPs by linkage disequilibrium analysis with the criteria of pairwise *r* ^2^ of ≥0.8 from the top 100 SNPs in 37 genomic regions and analyzed them using 2,783 Japanese nephrolithiasis cases and 5,251 controls. Fifty nine SNPs among 64 SNPs passed the same quality-control filtering as GWAS and were subjected to association analysis with nephrolithiasis by Cochran-Armitage trend test. We found 11 SNPs in five loci to be significantly associated with nephrolithiasis after Bonferroni correction (*P*<0.05/59 = 8.47×10^−4^, Table S3). A meta-analysis of GWAS and stage 2 revealed that seven SNPs cleared the genome wide significant threshold (*P*<5×10^−8^) (Table S4). Therefore, we considered stage 3 as a replication analysis for these seven SNPs.

Subsequently, we genotyped 11 SNPs in the additional cohort consisting of 2,109 nephrolithiasis cases and 4,622 controls. We excluded SNP rs10866504 from further analysis due to departure from Hardy-Weinberg equilibrium (HWE) in control samples. Five SNPs among ten cleared the significant threshold (P<0.005) in stage 3 (Table S5a). A meta-analysis with a fixed-effects model revealed all five SNPs indicated the significant association (*P*<5×10^−8^) without heterogeneity between three studies (Table 1). SNP rs3765623 also exhibited trend of association in stage 3 with p-value of 0.0371, however this SNP did not clear the genome wide significant threshold in the meta-analysis (P = 1.28×10^−7^) (Table S5b). Although SNP rs7981733 and rs1170155 also cleared the significant threshold in the meta-analysis (P = 1.43×10^−8^ and 3.89×10^−9^, respectively), these two SNPs did not indicate significant association in stage 3 (P = 0.0705 and 0.132, respectively) and exhibited heterogeneity between three studies (Table S5b). Therefore, further analysis is necessary to elucidate the role of these SNPs in the pathogenesis of nephrolithiasis. After all, we found three novel loci for nephrolithiasis: *RGS14-SLC34A1-PFN3-F12* on 5q35.3 (rs11746443; *P* = 8.51×10^−12^, odds ratio (OR) = 1.19), *INMT-FAM188B-AQP1* on 7p14.3 (rs1000597; *P* = 2.16×10^−14^, OR = 1.22), and *DGKH* on 13q14.1 (rs4142110; *P* = 4.62×10^−9^, OR = 1.14).

**Table 1:** Summary of GWAS and replication analyses.

SNP	stage	allele	gene	Case MAF^a^	Control MAF^a^	*P* b	OR^c^	95%CI^c^	*P*_het_ e
rs12654812	GWAS	a/g	*RGS14-SLC34A1-PFN3-F12*(5q35.3)	0.397	0.346	1.98×10^−5^	1.24	1.13–1.37	
	Stage2			0.381	0.354	5.57×10^−4^	1.13	1.05–1.20	
	Stage3/Replication			0.386	0.35	5.27×10^−5^	1.17	1.08–1.26	
	Combined^d^					4.42×10^−11^	1.16	1.11–1.22	0.267
rs11746443	GWAS	t/c	*RGS14-SLC34A1-PFN3-F12*(5q35.3)	0.29	0.244	1.62×10^−5^	1.27	1.14–1.41	
	Stage2			0.283	0.252	1.93×10^−5^	1.17	1.09–1.26	
	Stage3/Replication			0.278	0.25	4.64×10^−4^	1.16	1.07–1.26	
	Combined^d^					8.51×10^−12^	1.19	1.13–1.24	0.378
rs12669187	GWAS	t/c	*INMT-FAM188B-AQP1*(7p14.3)	0.223	0.176	1.04×10^−6^	1.34	1.19–1.51	
	Stage2			0.214	0.184	4.56×10^−6^	1.21	1.11–1.31	
	Stage3/Replication			0.205	0.182	1.59×10^−3^	1.16	1.06–1.27	
	Combined^d^					1.48×10^−12^	1.22	1.15–1.28	0.157
rs1000597	GWAS	g/a	*INMT-FAM188B-AQP1*(7p14.3)	0.246	0.202	1.06×10^−5^	1.29	1.15–1.45	
	Stage2			0.241	0.204	4.60×10^−8^	1.24	1.15–1.34	
	Stage3/Replication			0.23	0.203	5.43×10^−4^	1.17	1.07–1.28	
	Combined^d^					2.16×10^−14^	1.22	1.16–1.29	0.37
rs4142110	GWAS	a/g	*DGKH*(13q14.1)	0.41	0.46	7.15×10^−5^	1.22	1.11–1.35	
	Stage2			0.43	0.458	7.03×10^−4^	1.12	1.05–1.19	
	Stage3/Replication			0.431	0.459	2.18×10^−3^	1.12	1.04–1.20	
	Combined^d^					4.62×10^−9^	1.14	1.09–1.19	0.301

5,796 (904 in GWAS, 2,783 in stage2 and 2,109 in stage 3/replication) Nephrolithiasis cases and 17,344 (7,471 in GWAS and 5,251 in stage2 and 4,622 in stage3/replication) controls were analyzed.

a MAF: minor allele frequency.

b *P* value obtained from Cochrane-Armitage trend test.

c Odds ratios (OR) and confidence interval (CI) are calculated using the non-susceptible allele as reference.

d Combined: Odds ratio and *P* value for independence test were calculated by Mendel-hauzen and Laird method in the Meta-analysis.

e The *P* values of heterogeneities (*P*_het_) across three stages examined by using the Breslow-Day test.

Multiple SNPs in 5q35 and 7p14 regions indicated the significant association with nephrolithiasis. SNP rs11746443 in 5q35 was in strong linkage disequilibrium with rs12654812 (*D'* = 0.992 and *r^2^* = 0.600), while SNP rs1000597 in 7q14 was also in high linkage disequilibrium with rs12669187 (*D'* = 0.87 and *r^2^* = 0.64). To further evaluate the effects of these variations, we conducted the conditional analyses. As a result, all four SNPs remained to be significantly associated with nephrolithiasis (*P*<0.05, Table S6). These findings suggested that haplotypes carrying these SNPs would be associated with the risk of nephrolithiasis.

Then we selected the most significant SNPs from each of the three genomic region and examined the association of these SNPs with several clinical parameters [10], [11] which were shown to increase the risk of nephrolithiasis using up to a total of 27,323 independent Japanese samples [12]. We found no significant association of these SNPs with serum calcium, phosphorous, urate, and body mass index (BMI), but the risk allele of rs11746443 was significantly associated with the reduction of estimated glomerular filtration rate (eGFR) (*P* = 6.54×10^−8^, Table 2). We also conducted subgroup analyses stratified by gender, age, and BMI status, and found that SNP rs11746443 exhibited stronger effect among individuals with higher BMI (>24, OR of 1.27) than those with lower BMI (<24, OR of 1.14). However this difference (P = 0.04) was not statistically significant after the multiple testing correction (Figure S5).

**Table 2:** QTL analysis for serum phosphorus, serum calcium, serum urate, eGFR, and BMI.

Traits	SNP	MAF	beta	s.e.^a^	*P* b
Serum phosphorus					
	rs11746443	0.233	−0.074	0.35	8.32×10^−1^
	rs1000597	0.212	0.401	0.34	2.33×10^−1^
	rs4142110	0.435	−0.49	0.28	8.01×10^−2^
Serum calcium					
	rs11746443	0.233	0.00049	0.019	9.80×10^−1^
	rs1000597	0.204	−0.0019	0.019	3.24×10^−1^
	rs4142110	0.464	0.025	0.016	1.11×10^−1^
Serum urate					
	rs11746443	0.231	−0.0032	0.02	8.75×10^−1^
	rs1000597	0.214	0.012	0.02	5.72×10^−1^
	rs4142110	0.344	−0.0067	0.016	6.81×10^−1^
estimated glomerular filtration rate (eGFR)					
	rs11746443	0.23	−1.85	0.34	6.54×10^−8^
	rs1000597	0.211	0.176	0.33	5.97×10^−1^
	rs4142110	0.461	0.33	0.27	2.24×10^−1^
Body mass index (BMI)					
	rs11746443	0.23	0.096	0.04	1.71×10^−2^
	rs1000597	0.211	0.074	0.039	5.01×10^−2^
	rs4142110	0.461	−0.00011	0.032	9.97×10^−1^

QTL analysis for serum level for phosphorus (n = 2,085), calcium (n = 4,807), urate (n = 16,702), eGFR (n = 21,842), and BMI (n = 24,406). Estimated GFR (eGFR) is calculated by formulas using blood test result, age, gender. eGFR (mL/min/1.73 m^2^) = 194×serum creatinine (mg per 100 ml)^−1.094^×age−^0.287^ (×0.739 if female).

a s.e. = standard error of mean,

b *P* values were obtained from logistic regression analysis using age, gender, smoking, alcohol, and BMI (phosphorus, calcium, and urate); smoking, alcohol, and BMI (eGFR); or age, gender, smoke, and alcohol (BMI) as covariates.

**Figure 1:**
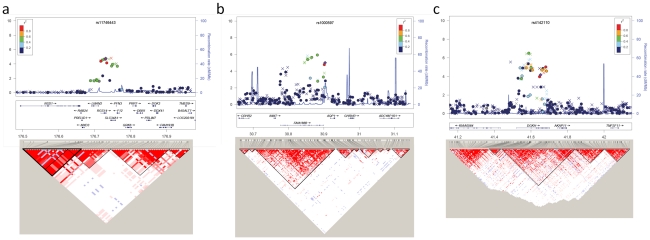
Regional association plots at rs11746443, rs1000597, and rs4142110 loci. (a–c) Upper panel; *P*-values of genotyped SNPs (circle) and imputed SNPs (cross) are plotted (as −log_10_ *P*-value) against their physical position on chromosome 5 (a), 7 (b), and 13(c) (NCBI Build 36). SNPs rs11746443 on 5q35 (a), rs1000597 on 7p14 (b), and rs4142110 on 13q14 (c) are represented by purple diamonds. The genetic recombination rates estimated from 1000 Genomes samples (JPT+CHB) are shown with a blue line. SNP's color indicates LD with rs11746443 (a), rs1000597 (b), and rs4142110 (c) according to a scale from *r* ^2^ = 0 to *r* ^2^ = 1 based on pair-wise *r* ^2^ values from HapMap JPT. Middle Panel; Gene annotations from the University of California Santa Cruz genome browser. Lower Panel; We drew the LD map based on *D*' values using the genotype data of the cases and controls in the GWAS samples.

To further characterize these loci, we conducted imputation analyses of each genomic region in the GWAS samples (904 cases and 7,471 controls) using data from HAPMAP phase II (JPT). The regional association plots revealed that all modestly-associated SNPs are confined within an *RGS14-SLC34A1-PFN3-F12* region on chromosome 5q35.3, an *INMT-FAM188B-AQP1* region on 7p14.3, and a *DGKH* region on 13q14.1, respectively (Figure 1a, 1b, 1c and Figure S6a, S6b, S6c). The result of imputation analysis revealed that a SNP rs3812036 in intron 4 of *SLC34A1* showed the strongest association with *P*-value of 1.48×10^−5^ among SNPs within the *RGS14-SLC34A1-PFN3-F12* region (Figure S6a and Table S7). *SLC34A1* encodes NPT2, a member of the type II sodium-phosphate co-transporter family which was highly expressed in kidney (Figure S7a). Mutations of *SLC34A1* were known to cause hypophosphatemic nephrolithiasis and osteoporosis in human [13] and severe renal phosphate wasting and hypercalciuria in mice [14]. In addition, a GWAS reported previously revealed the association of variations on *SLC34A1* locus with kidney function [15] and serum phosphorus concentration [16]. Although we did not find a significant association with serum phosphorus in Japanese population, the risk allele of SNP rs11746443 was associated with the reduction of eGFR, a marker of renal function, suggesting that variations in this region could regulate renal function and subsequently affect the risk of nephrolithiasis.

The most significantly-associated SNP rs1000597 on chromosome 7p14.3 is located at 5.2 kb downstream of the *FAM188B* gene and at 14.2 kb upstream of the *AQP1* gene which encodes aquaporin-1 (Figure S8a). The result of imputation analysis revealed that more than ten strongly associated SNPs were clustered around *FAM188B* region (Table S8a), however the role of FAM188B in the pathogenesis of nephrolithiasis was not reported so far. Aquaporin-1 was abundantly expressed in kidney (Figure S7b) and function as a water channel [17]. Moreover, *Aqp1* null mice exhibited reduced osmotic water permeability in membrane of kidney proximal tube and became severely dehydrated after water deprivation, indicating the important role for aquaporin-1 in the urinary concentrating mechanism [17]. Interestingly, rs1000597 is also located within intron 6 of ENST00000434909, which is predicted to encode a 329-amino-acid protein consisting of the carboxy-terminal portion of FAM188B (residue 655–742) and the carboxy-terminal portion of aquaporin-1 (residue 29–269) (Figure S8b). Although the physiological roles of ENST00000434909 have not been characterized yet, quantitative real-time PCR analysis revealed that this gene was preferentially expressed in kidney (Figure S8c). Taken together, SNP rs1000597 is likely to be associated with regulation of *AQP1* and/or ENST00000434909 expression, and subsequently affect the urine-concentration process and increase the risk of nephrolithiasis.

SNP rs4142110 on *DGKH* was also indicated strong association by the meta-analysis of three studies. *DGKH* was expressed in brain (Figure S7c) and possibly elated to psychiatric disorders such as bipolar and major depressive disease [18], [19], but its involvement in renal function or calcium homeostasis has not been reported. Although several SNPs on chromosome 13p14.1 including rs7981733 showed stronger association with nephrolithiasis than rs4142110 in GWAS and imputation analysis (Table S8b), none of these associated SNPs alter amino acid sequence. Therefore, further association and functional analyses would be essential to elucidate the role of this variation in the etiology of nephrolithiasis.

The *CLDN14* gene was shown to be associated with nephrolithiasis in the previous GWAS in Caucasian [8], but SNPs rs219778 and rs219781 did not exhibit significant association (P = 0.937 and 0.630, respectively), while SNP rs219780 was not polymorphic in Japanese. However SNP rs2835349 which is located at 19 kb upstream of the *CLDN14* gene was included in the top 100 SNPs in our GWAS (*P* = 6.33×10^−5^ with OR of 1.22, Figure S9). SNP rs2835349 did not achieve the threshold of the first replication analysis (*P*<8.47×10^−4^) but revealed some trend of the association with *P*-value of 3.29×10^−3^ and OR of 1.10 (Table S9). To further investigate the association of this variation with nephrolithiasis, we genotyped rs2835349 using stage 3 cohort. Although rs2835349 failed to show the replication of association with *P*-value of 0.624 and OR of 0.98 (95% C.I. of 0.94–1.10), metanalysis of three studied indicated the suggestive association (*P* = 5.72×10^−4^ and OR of 1.10). Taken together, *CLDN14* would be a common genetic locus for nephrolithiasis in both Caucasian and Japanese populations.

Then we conducted the multiple logistic regression analysis of three significant variations (rs11746443, rs1000597, and rs4142110) including age, gender, and BMI as covariates. As a result, these variations remained as factors that were strongly associated with nephrolithiasis with OR of 1.18 (95% confidence interval (C.I.) of 1.12–1.25), 1.21 (95% C.I. of 1.14–1.27), and 1.14 (95% C.I. of 1.09–1.19), respectively (Table S10), indicating these SNPs as an independent risk factors. Therefore, we examined cumulative effect of these variations using weighted genetic risk score (wGRS) as describe previously [20]. We defined 4 categories by using the mean and SD of control samples (Group 1: less than −1S.D. (<0.127), Group 2: between −1S.D. and mean (0.127–0.309), Group 3 between mean and +1S.D. (0.309–0.490), Group 4: more than +1S.D. (0.490<)). As a result, we found that individuals in Group 4 have 1.95-fold higher risk of nephrolithiasis than those in Group 1 (Table S11a, S11b). These results indicated the cumulative effect of these variations.

Through the GWAS and two sets of replication studies using a total of 5,892 cases and 17,809 controls, we identified three significantly-associated loci for nephrolithiasis. Although the molecular mechanisms how these variations could increase the risk of nephrolithiasis should be further investigated, our results elucidated crucial roles of genetic factors related with renal function as well as pathogenesis of nephrolithiasis. Nephrolithiasis is considered as one of lifestyle-related diseases; low fluid intake, low dietary calcium, and high dietary salt have been shown to increase the disease risk. However the results of dietary intervention studies to reduce the recurrence incidence of nephrolithiasis has been unsuccessful [21]. We think that our findings would contribute to the better understanding of pathogenesis of nephrolithiasis and lead to the development of new innovative therapeutics.

## Materials and Methods

### Ethics statement

All subjects provided written informed consent. This project was approved by the ethical committees at the Institute of Medical Science, University of Tokyo, and Center for Genomic Medicine, Institutes of Physical and Chemical Research (RIKEN).

### Samples

Characteristics of each cohort are shown in Table S1. In this study, we conducted GWAS and two subsequent replication analyses using a total of 5,892 nephrolithiasis cases and 17,809 control subjects. All case samples and 16786 of non-nephrolithiasis case-mix control subjects were obtained from BioBank Japan project, “the Leading Project for Personalized Medicine” in the Ministry of Education, Culture, Sports, Science and Technology [22]. We excluded patients with struvite, cystine, ammonium acid urate, and uric acid stone. Patients with secondary nephrolithiasis caused by drugs, hyperparathyroidism, or congenital anomalies of urinary tract were also excluded. We excluded the subjects with a history of diabetes, hypertension, and dyslipidemia from controls, because these diseases were shown to be a risk factor for nephrolithiasis [23]. We also obtained 1023 Japanese control DNAs from healthy volunteers from the Osaka-Midosuji Rotary Club, Osaka, Japan. A total of 27,323 Japanese samples from Biobank Japan were used for the QTL analyses of serums calcium, phosphorus, urate, BMI and eGFR.

### SNP genotyping

Genomic DNA was extracted from peripheral blood leukocytes using a standard method. In the GWAS, a total of 1000 cases and 7,936 non-nephrolithiasis controls (cerebral aneurysm, primary sclerosing cholangitis, esophageal cancer, uterine body cancer chronic obstructive pulmonary, glaucoma, and healthy volunteers) were genotyped at 712,726 SNPs using Human Omni Express (Figure S1). We performed a standard quality control procedure to exclude SNPs with low call rate (<99%), *P*-value of Hardy-Weinberg equilibrium test of <1.0×10^−6^ in controls, and minor allele frequency (MAF) of <0.01 in each stage. Finally, we analyzed 556,249 SNPs in GWAS. In stage2, we genotyped 2,783 cases and 5,251 non-nephrolithiasis controls (epilepsy, atopic dermatitis, and Grave's disease) by using multiplex PCR-based Invader assay (Third Wave Technologies) or Human Omni Express, respectively. In replication/stage 3, we genotyped 2,109 cases and 4,622 non-nephrolithiasis controls (chronic hepatitis C, liver cancer, and colon cancer) using multiplex PCR-based Invader assay (Third Wave Technologies). For the QTL analyses, we genotyped 27,323 samples using Illumina HumanHap610-Quad Genotyping BeadChip as described previously [24].

### Statistical analysis

The association of SNPs with nephrolithiasis in each stage was tested by a 1-degree-of-freedom Cochran-Armitage trend test using PLINK [25]. The genomic inflation factor λ was calculated using all the tested SNPs in the GWAS. The quantile-quantile plot was drawn using R program. The Odds ratios were calculated using the non-susceptible allele as reference, unless it was stated otherwise elsewhere. The combined analysis of GWAS and the replication stages was performed utilizing the Mantel-Haenszel method. In this study, we set genome wide significance threshold of 5×10^−8^ in meta-analysis of all stages [26], [27]. We also assumed a significant level of 7.85×10^−5^ (TOP 100 SNPs) in GWAS, 8.47×10^−4^ (0.05/59) in stage2, and 5.00×10^−3^ (0.05/10) in stage 3, respectively. We selected SNPs which satisfied all these four criteria. Heterogeneity across three stages was examined by using the Breslow-Day test [28]. The statistical power is 31.6% in GWAS (P = 7.85×10^−5^), 96.38% in stage2 (P = 0.05/59), 95.54% in stage3 (P = 0.05/10), and 99.37% in all stage (P = 5.00×10^−8^) at minor allele frequency of 0.3 and Odd ratio of 1.2. For multiple logistic regression analysis at rs11746443, rs1000597 and rs4142110, we considered age at recruitment, gender, and BMI as covariates using the R program.

### Imputation analysis

We used a Hidden Markov model programmed in MACH [29] and haplotype information from HapMap JPT samples to infer the genotypes of untyped SNPs in the GWAS. We applied the same SNP quality criteria as in GWAS for selecting SNPs for the analysis. The association was tested by 1-degree-of-freedom Cochran-Armitage trend test.

### Serum calcium, phosphorus, urate, BMI, and a kidney functional index of eGFR analysis

We assessed the effect of genetic variations on serum calcium, phosphorus, urate, BMI, and eGFR, as described previously [30]. In brief, we used the genotyping result from 27,323 Japanese individuals with various diseases those were genotyped by using the Illumina Human610-Quad BeadChip. Since three significant SNPs (rs11746443, rs1000597, and rs4142110) were not included in Illumina Human610-Quad BeadChip, genotype data of these SNPs were determined by imputation using the MACH software. We compared the data from imputation analysis and that form direct sequencing for SNPs rs11746443, rs1000597 and rs4142110 using 94 samples. The concordance rates were 100% for rs1000597 and rs4142110 and 97.7% for rs11746443. All the samples were obtained from Biobank Japan. For serum calcium, phosphorus and urate, a linear regression model was used to analyze the associations of quantitative traits with SNP genotypes by incorporating age, gender, alcohol, smoking status, and BMI as covariates. For BMI, a linear regression model was used to analyze the associations of quantitative traits with SNP genotypes by incorporating age, gender, alcohol, smoking status as covariates. GFR was estimated using the following formula [31]: eGFR (mL/min/1.73 m^2^) = 194×serum creatinine (mg per 100 ml)^−1.094^×age−^0.287^ (×0.739 if female). For eGFR, a linear regression model was used to analyze the associations of quantitative traits with SNP genotypes by incorporating alcohol, smoking status, as covariates.

### Quantitative real-time PCR

mRNA or total RNA from normal tissues were purchased from Calbiochem and BioChain. cDNAs were synthesized with the SuperScript Preamplification System (Invitrogen). Quantitative real-time PCR was conducted using the SYBR Green I Master on a LightCycler 480 (Roche). The primer sequences are indicated in Table S12. ENST00000434909 cDNA was amplified with a primer pair encompassing the region containing from exon 5 to exon7. Gene structure of ENST00000434909 was confirmed by direct sequencing of PCR product.

### The weighted Genetic Risk Score (wGRS)

The wGRS was calculated by multiplying the number of risk alleles for each SNP by weight for that SNP (the natural log of the odds ratio for each allele) (Table S11a), and then taking the sum across the 3 SNPs according to the following formula:
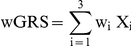
Where i is the SNP, w_i_ is the weight for SNP_i_, and X_i_ is the number of risk alleles (0, 1 or 2).

### Software

For general statistical analysis, we employed R statistical environment version 2.9.1 (cran.r-project.org) or plink-1.06 (pngu.mgh.harvard.edu/∼purcell/plink/). The Haploview software version 4.2 [32] was used to calculate LD and to draw Manhattan plot. Primer3 -web v0.3.0 (http://frodo.wi.mit.edu) web tool was used to design primers. We employed LocusZoom (http://csg.sph.umich.edu/locuszoom/) for plotting regional association plots. We used SNP Functional Prediction [33] web tool for functional annotation of SNPs (http://snpinfo.niehs.nih.gov/snpfunc.htm). We used MACTH [34] web tool for searching potential binding sites for transcription factors (http://www.gene-regulation.com/index.htm).

## Supporting Information

Figure S1 Summary of study design and results.
(TIF)

Figure S2 Principal Component Analysis (PCA) plots showing the distribution of case and control used in the GWAS. CEU; Utah residents with Northern and Western European ancestry from the CEPH collection. YRI; Yoruban in Ibadan, Nigeria. CHB; Han Chinese in Beijing, China. JPT; Japanese in Tokyo, Japan. Case and control samples belonged to Hondo cluster according to PCA were used in this study.
(TIF)

Figure S3 Association analysis using all samples included in Hondo and Ryukyu clusters. Quantile-quantile plots for test statistics (Cochran-Armitage trend test) for 556,249 SNPs passing the quality control. Black dots are the uncorrected test statistics (λ = 1.123). Under the null hypothesis of no association at any loci, the points would be expected to follow the red line (y = x).
(TIF)

Figure S4 a) Association analysis using all samples. Quantile-quantile plots for test statistics (logistic regression analysis using top principle components as covariate) for 556,249 SNPs passing the quality control. Black dots are the uncorrected test statistics (λ = 1.054). Under the null hypothesis of no association at any loci, the points would be expected to follow the red line (y = x). b, Association analysis using samples included in a Hondo cluster. Quantile-quantile plots for test statistics (Cochran-Armitage trend test) for 556,249 SNPs passing the quality control. Black dots are the uncorrected test statistics (λ = 1.042). Under the null hypothesis of no association at any loci, the points would be expected to follow the red line (y = x). c, Association analysis using samples included in a Hondo cluster. Manhattan plot showing the genome-wide *P* values of association. The *P* values were calculated by Cochran-Armitage trend test. The y axis represents the −log10 *P* values of 556,249 SNPs, and x axis shows their chromosomal positions. The horizontal blue line shows the threshold of P≤7.85×10^−5^ for selecting top 100 SNPs for replication.
(PDF)

Figure S5 Forest plots for stratified association analysis at rs11746443 (a), rs1000597 (b) and rs4142110 (c). Case and control samples from GWAS, replication 1, and replication 2 were stratified by age (case; ≥60 n = 2006 <60 n = 3689, control; ≥60 n = 8300 <60 n = 8837), gender (case; male n = 4268 female n = 1434, control; male n = 9462 female n = 7863), and BMI (case; ≥24 n = 2592 <24 n = 2656, control; ≥24 n = 4781 <24 n = 10586). Odds ratios and confidence intervals are calculated from fixed effect model implemented in R. The *P* values of heterogeneities (*P*_het_) across three stages examined by using the Breslow-Day test.
(PDF)

Figure S6 Enlarged regional plots of three significant susceptibility loci at 5q35, 7p14, and 13q14. (a–c) Upper panel; *P* values of genotyped SNPs (circle) and imputed SNPs (cross) are plotted (as −log_10_ *P* value) against their physical position on chromosome 5 (a), 7 (b), and 13(c) (NCBI Build 36). SNPs rs11746443 on 5q35 (a), rs1000597 on 7p14 (b), and rs4142110 on 13q14 (c) are represented by purple diamonds. The genetic recombination rates estimated from 1000 Genomes samples (JPT+CHB) are shown with a blue line. SNP's color indicates LD with rs11746443 (a), rs1000597 (b), and rs4142110 (c) according to a scale from *r* ^2^ = 0 to *r* ^2^ = 1 based on pair-wise *r* ^2^ values from HapMap JPT. Middle Panel; Gene annotations from the University of California Santa Cruz genome browser. Lower Panel; We drew the LD map based on *D*' values using the genotype data of the cases and controls in the GWAS samples.
(PDF)

Figure S7 Expression of *SLC34A1*, *AQP1* and *DGKH* genes in 27 normal tissues. Quantitative PCR analysis of *SLC34A1* (a), *AQP1* (b), and *DGKH* (c) in normal tissues. *ACTB* was used for normalization of expression levels.
(TIF)

Figure S8 Expression of *ENST00000434909*. (a) Gene Structure of *FAM188B*, *AQP1* and *ENST00000434909*. Locations of rs12669187 and rs1000597 are intronic 4 and intronic 6 of *ENST00000434909*. (b) Protein structure of AQP1, FAM188B, and ENST00000434909. (c) Quantitative PCR analysis of *ENST00000434909* in normal tissues. ACTB was used for normalization of expression levels.
(TIF)

Figure S9 Regional association plots at rs2835349 loci. Upper panel; *P*-values of genotyped SNPs (circle) and imputed SNPs (cross) are plotted (as −log_10_ *P*-value) against their physical position on chromosome 21 (NCBI Build 36). SNPs rs2835349 on 21q22 are represented by purple diamonds. The genetic recombination rates estimated from 1000 Genomes samples (JPT+CHB) are shown with a blue line. SNP's color indicates LD with rs2835349 according to a scale from *r* ^2^ = 0 to *r* ^2^ = 1 based on pair-wise *r* ^2^ values from HapMap JPT. Middle Panel; Gene annotations from the University of California Santa Cruz genome browser. Lower Panel; We drew the LD map based on *D*' values using the genotype data of the cases and controls in the GWAS samples.
(TIF)

Table S1 Characteristics of samples and methods used in this study.
(DOCX)

Table S2 The Result of GWAS for nephrolithiasis (Top 100 SNPs).
(DOCX)

Table S3 Results of association analysis for nephrolithiasis in stage2 (59 SNPs).
(DOCX)

Table S4 Results of meta-analysis between GWAS and Stage 2 for nephrolithiasis (59 SNPs).
(DOCX)

Table S5 a) Results of association analysis for nephrolithiasis in stage3/replication (11 SNPs). b) Results of meta analysis of 3 stage (11SNPs).
(DOCX)

Table S6 The results of conditional analysis for significantly SNPs in 5q35.3 or 7p14.3 associated with nephrolithiasis in all stages.
(DOCX)

Table S7 Result of imputation analysis at 5q35.3.
(DOCX)

Table S8 Result of imputation analysis and snp location at 7p14.3 (a) and 13q14.1 (b).
(DOCX)

Table S9 Association of rs2835349 with nephrolithiasis in the all stage and combined analysis.
(DOCX)

Table S10 Multiple logistic regression analysis for risk of nephrolithiasis.
(DOCX)

Table S11 a) SNPs that compose the weighted genetic risk score and weights assigned to each marker. b) wGRS scores and odd ratios of nephrolithiasis by wGRS groups.
(DOCX)

Table S12 Sequences of primers.
(DOCX)
