# Supplementary figures and images for: A Genome-Wide Association Study of Nephrolithiasis in the Japanese Population Identifies Novel Susceptible Loci at 5q35.3, 7p14.3, and 13q14.1
Source: PLoS Genet. 2012 Mar 1;8(3):e1002541. doi: 10.1371/journal.pgen.1002541 (PMC3291538; doi:10.1371/journal.pgen.1002541)

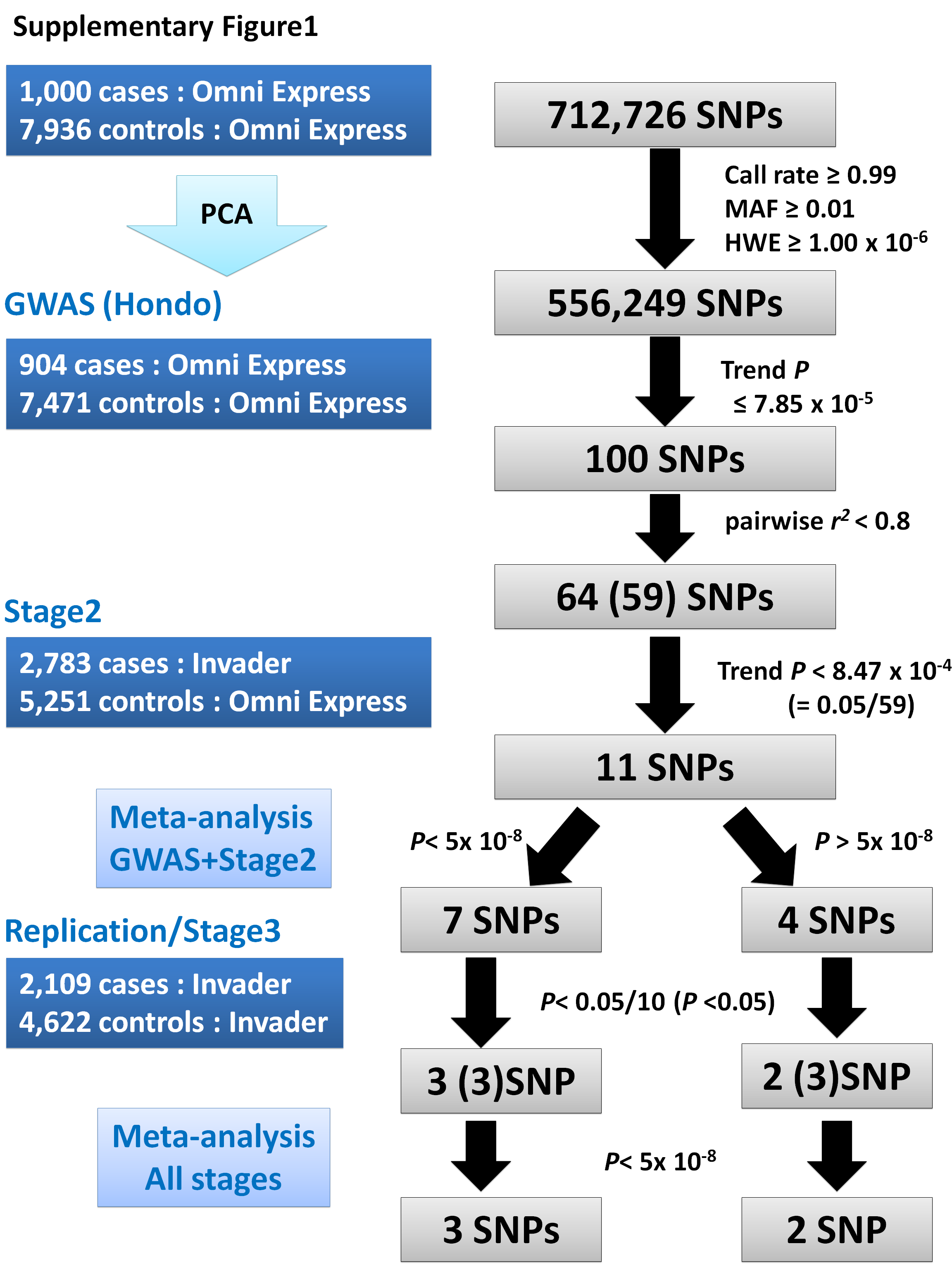

Supplement: Figure S1 — Summary of study design and results. (TIF) [file pgen.1002541.s001.tif]

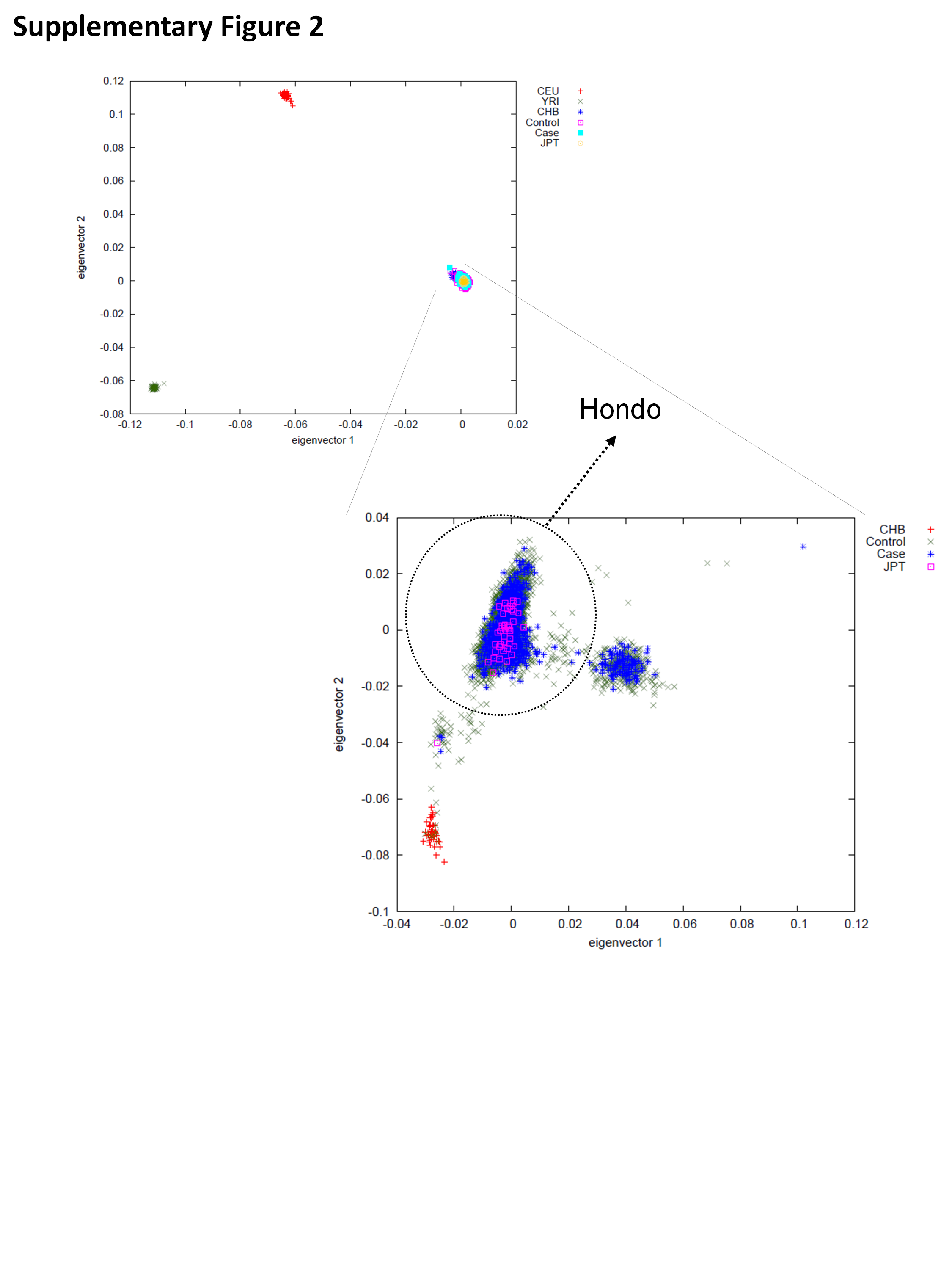

Supplement: Figure S2 — Principal Component Analysis (PCA) plots showing the distribution of case and control used in the GWAS. CEU; Utah residents with Northern and Western European ancestry from the CEPH collection. YRI; Yoruban in Ibadan, Nigeria. CHB; Han Chinese in Beijing, China. JPT; Japanese in Tokyo, Japan. Case and control samples belonged to Hondo cluster according to PCA were used in this study. (TIF) [file pgen.1002541.s002.tif]

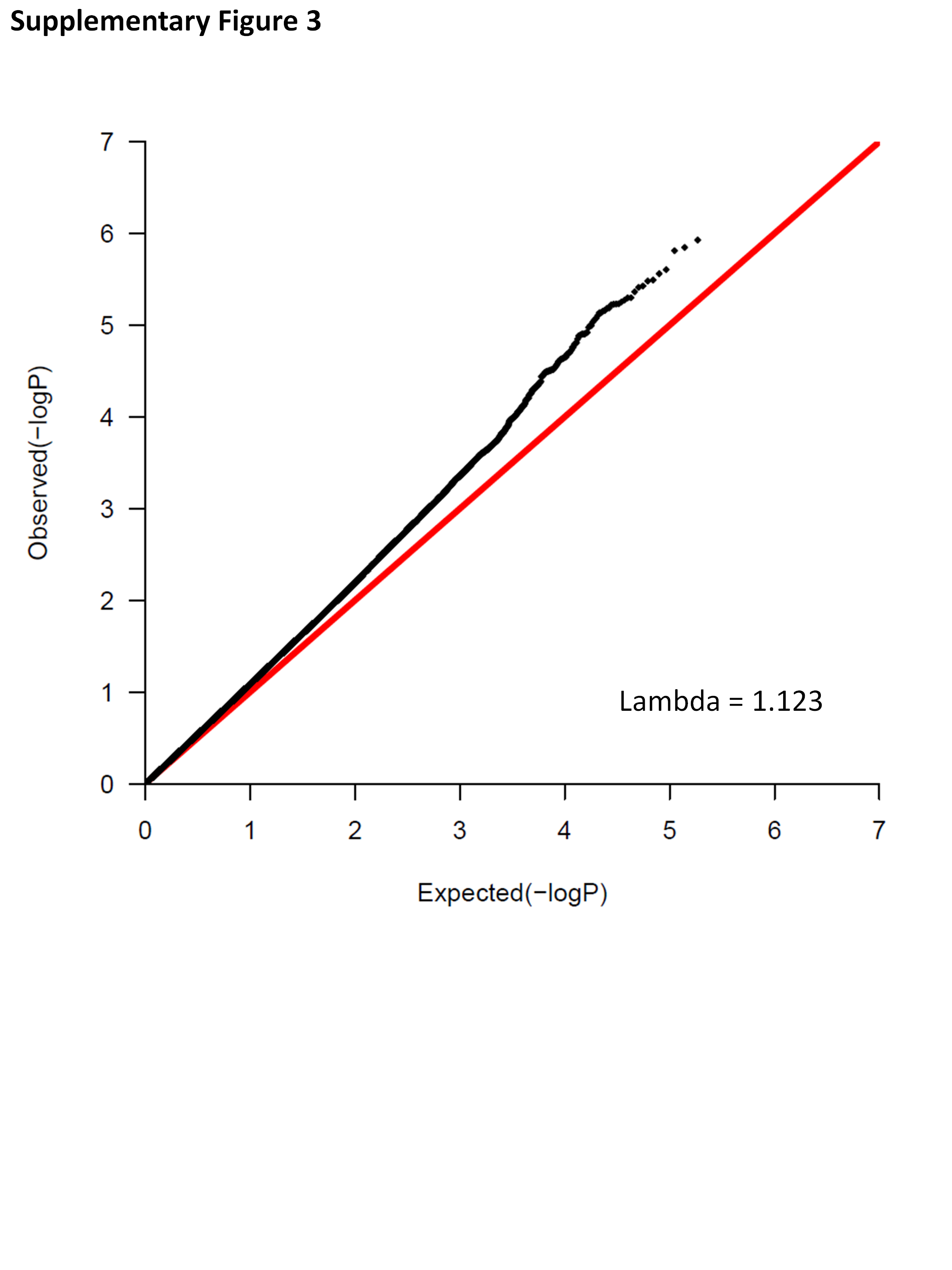

Supplement: Figure S3 — Association analysis using all samples included in Hondo and Ryukyu clusters. Quantile-quantile plots for test statistics (Cochran-Armitage trend test) for 556,249 SNPs passing the quality control. Black dots are the uncorrected test statistics (λ = 1.123). Under the null hypothesis of no association at any loci, the points would be expected to follow the red line (y = x). (TIF) [file pgen.1002541.s003.tif]

Supplementary Figure 4

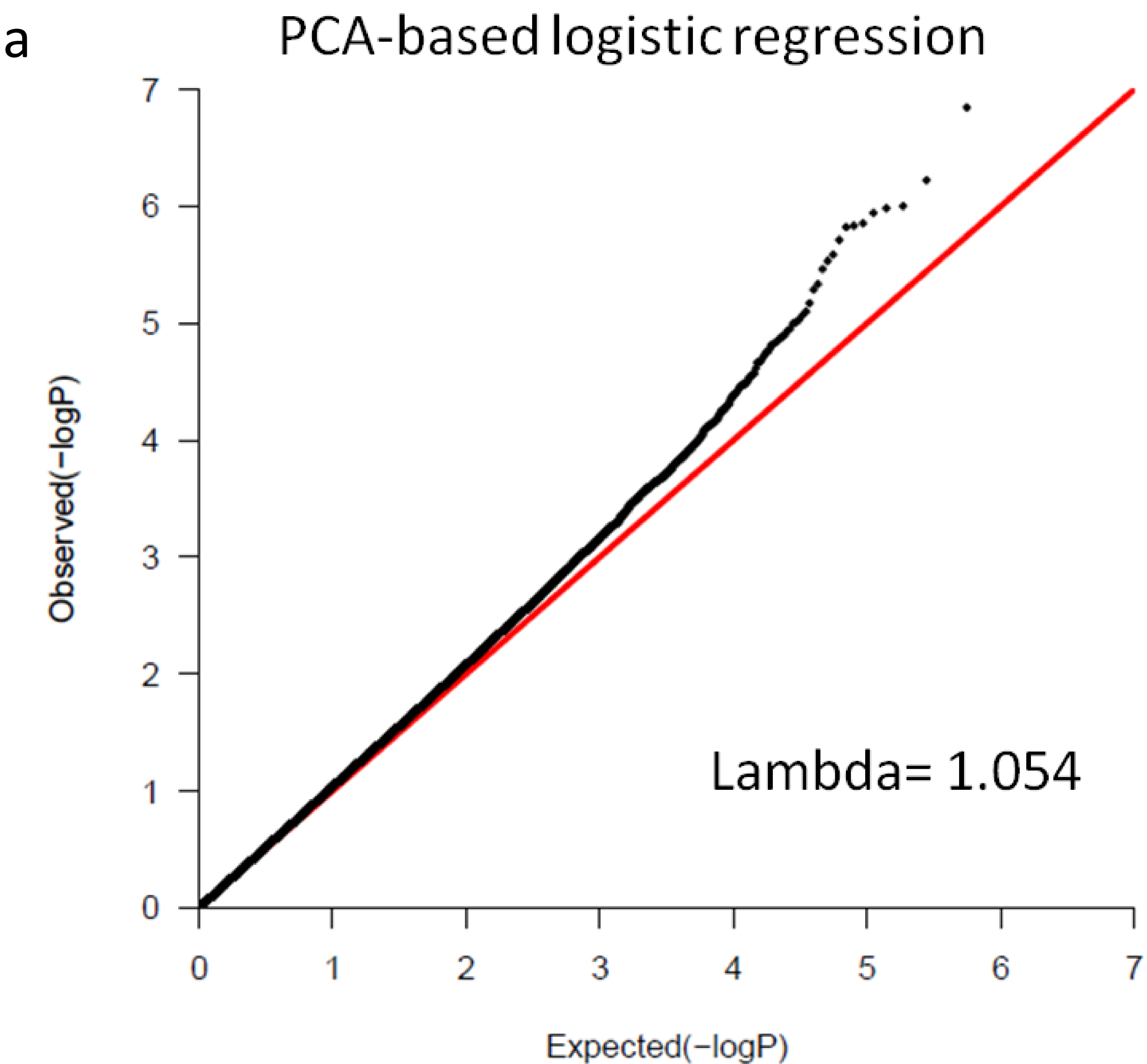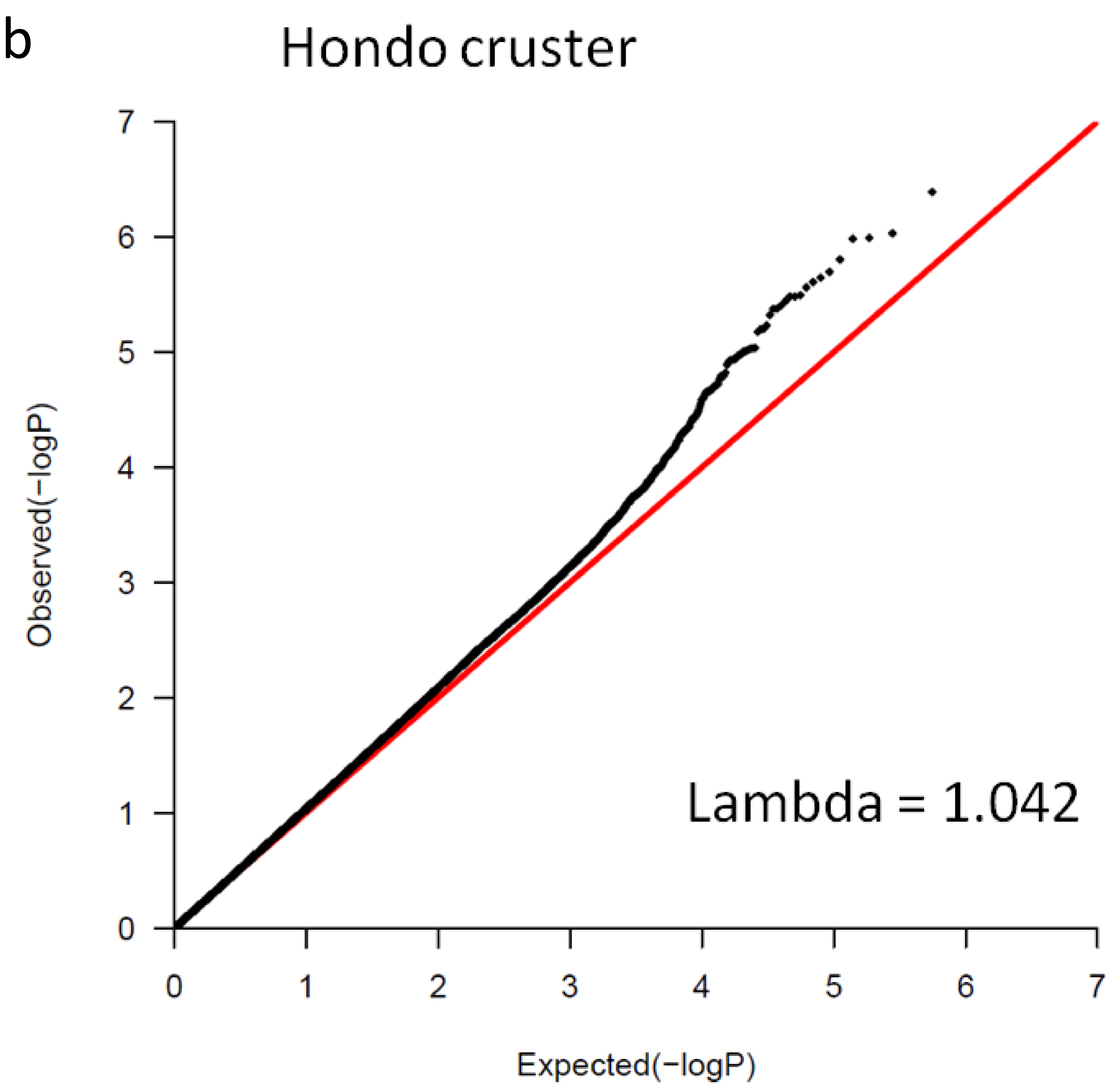

C

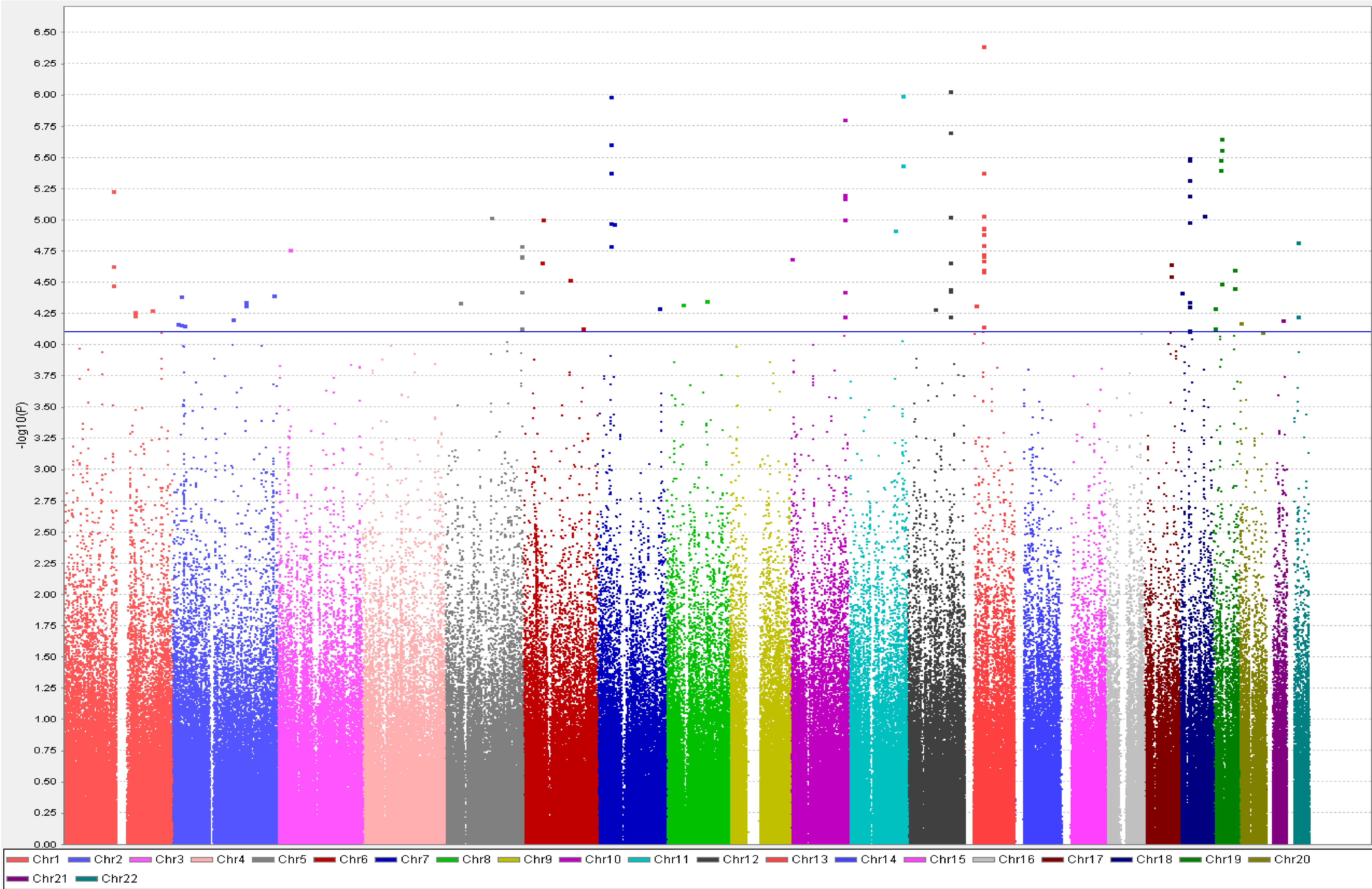

Supplement: Figure S4 — a) Association analysis using all samples. Quantile-quantile plots for test statistics (logistic regression analysis using top principle components as covariate) for 556,249 SNPs passing the quality control. Black dots are the uncorrected test statistics (λ = 1.054). Under the null hypothesis of no association at any loci, the points would be expected to follow the red line (y = x). b, Association analysis using samples included in a Hondo cluster. Quantile-quantile plots for test statistics (Cochran-Armitage trend test) for 556,249 SNPs passing the quality control. Black dots are the uncorrected test statistics (λ = 1.042). Under the null hypothesis of no association at any loci, the points would be expected to follow the red line (y = x). c, Association analysis using samples included in a Hondo cluster. Manhattan plot showing the genome-wide P values of association. The P values were calculated by Cochran-Armitage trend test. The y axis represents the −log10 P values of 556,249 SNPs, and x axis shows their chromosomal positions. The horizontal blue line shows the threshold of P≤7.85×10^−5^ for selecting top 100 SNPs for replication. (PDF) [file pgen.1002541.s004.pdf]

# Supplementary Figure 6

a

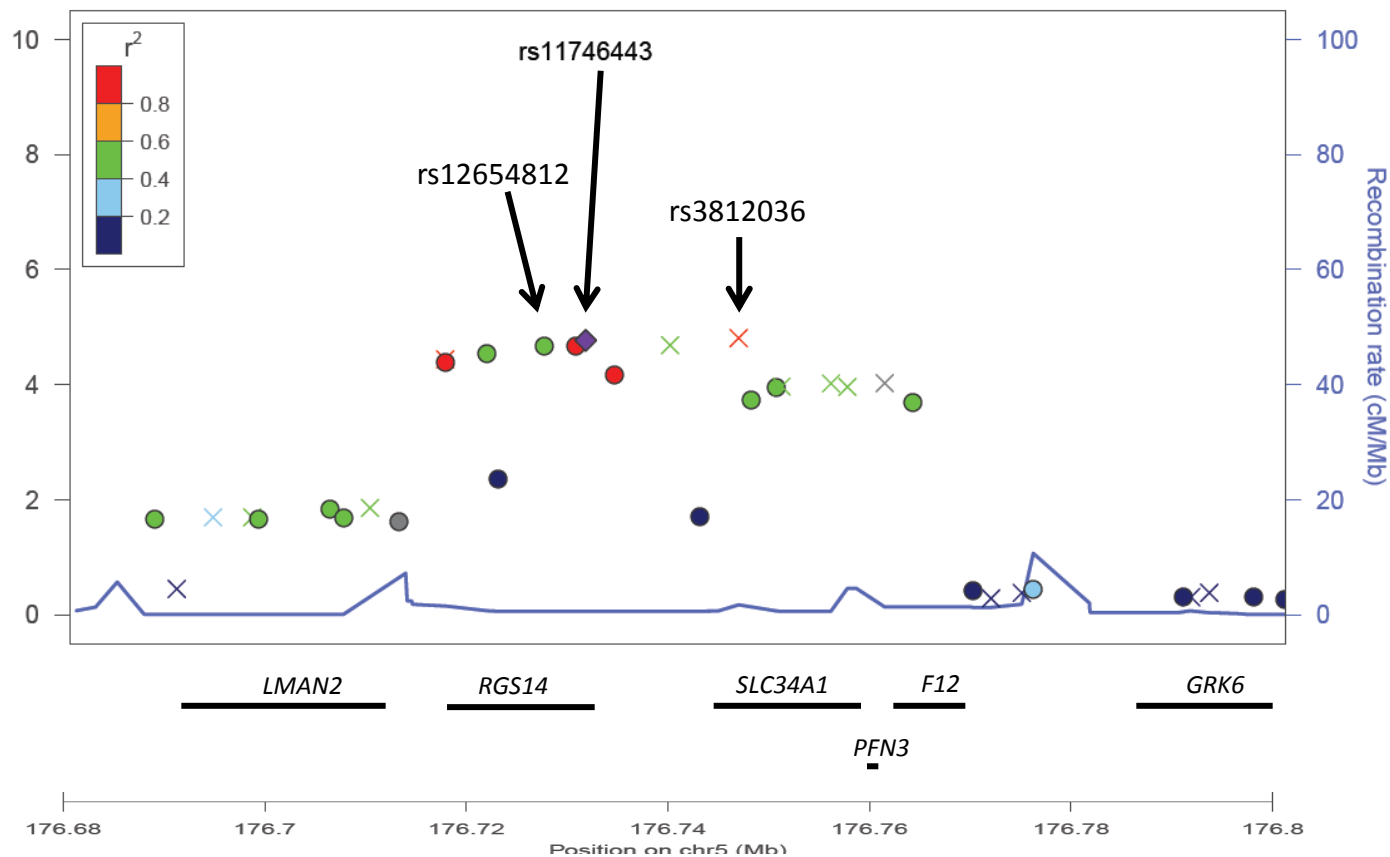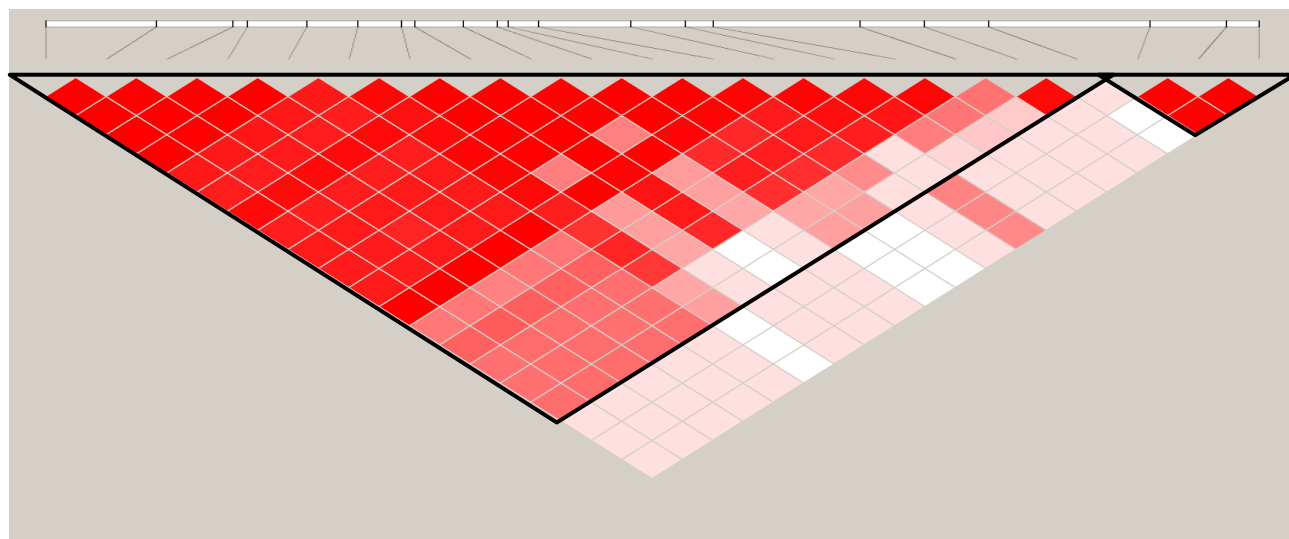

**b**

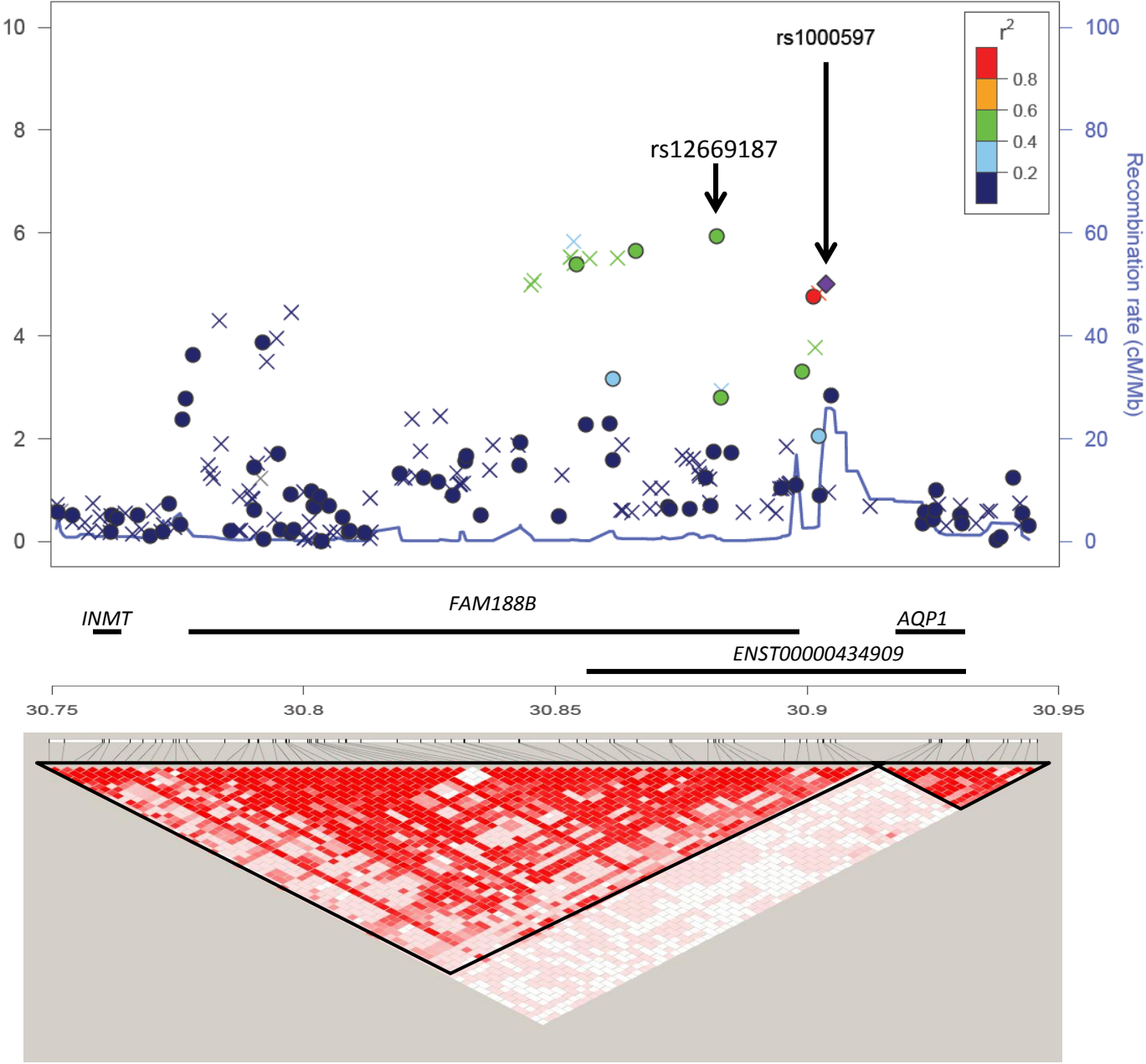

**c**

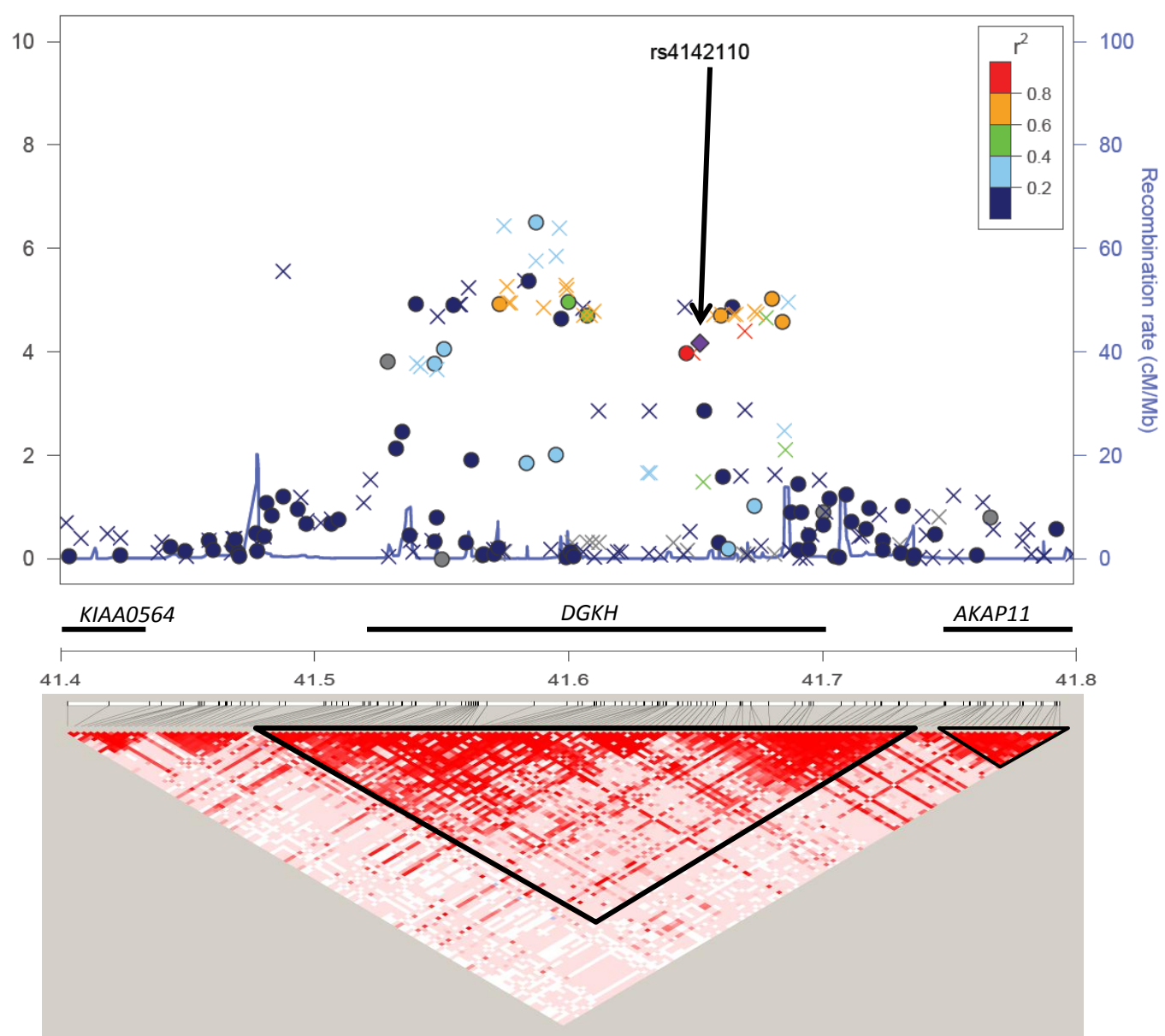

Supplement: Figure S6 — Enlarged regional plots of three significant susceptibility loci at 5q35, 7p14, and 13q14. (a–c) Upper panel; P values of genotyped SNPs (circle) and imputed SNPs (cross) are plotted (as −log10 P value) against their physical position on chromosome 5 (a), 7 (b), and 13(c) (NCBI Build 36). SNPs rs11746443 on 5q35 (a), rs1000597 on 7p14 (b), and rs4142110 on 13q14 (c) are represented by purple diamonds. The genetic recombination rates estimated from 1000 Genomes samples (JPT+CHB) are shown with a blue line. SNP's color indicates LD with rs11746443 (a), rs1000597 (b), and rs4142110 (c) according to a scale from r 2 = 0 to r 2 = 1 based on pair-wise r 2 values from HapMap JPT. Middle Panel; Gene annotations from the University of California Santa Cruz genome browser. Lower Panel; We drew the LD map based on D' values using the genotype data of the cases and controls in the GWAS samples. (PDF) [file pgen.1002541.s006.pdf]

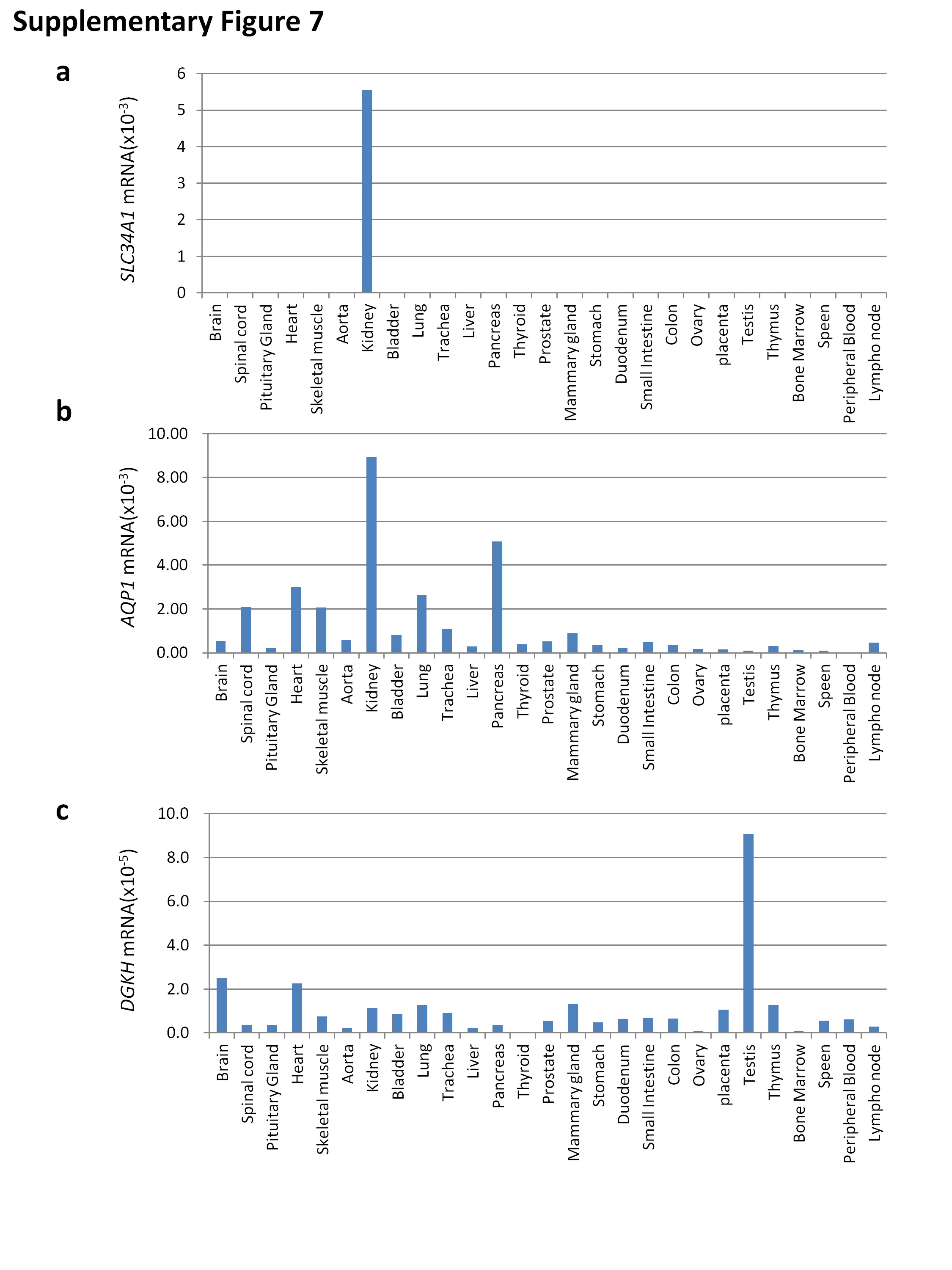

Supplement: Figure S7 — Expression of SLC34A1, AQP1 and DGKH genes in 27 normal tissues. Quantitative PCR analysis of SLC34A1 (a), AQP1 (b), and DGKH (c) in normal tissues. ACTB was used for normalization of expression levels. (TIF) [file pgen.1002541.s007.tif]

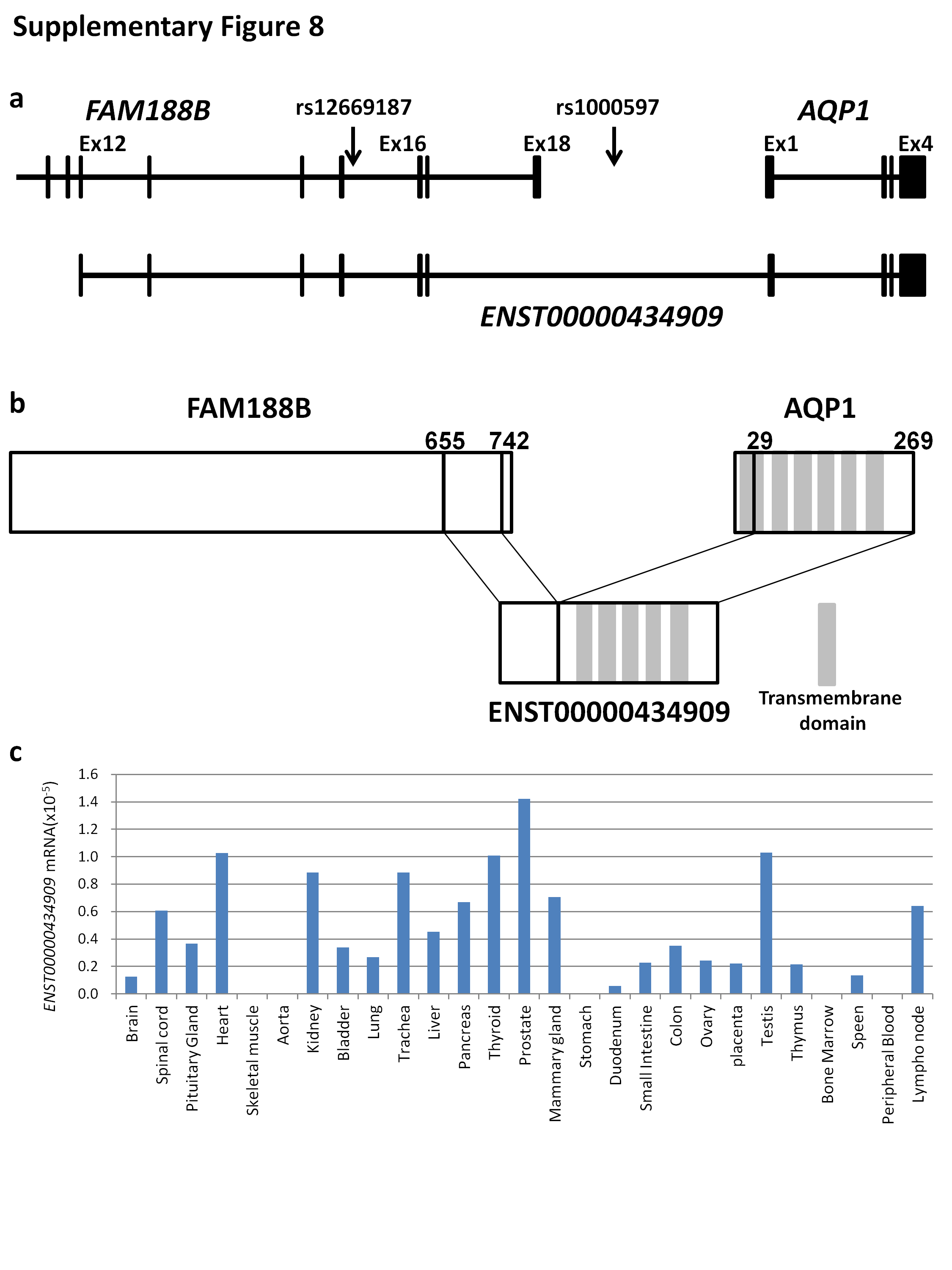

Supplement: Figure S8 — Expression of ENST00000434909. (a) Gene Structure of FAM188B, AQP1 and ENST00000434909. Locations of rs12669187 and rs1000597 are intronic 4 and intronic 6 of ENST00000434909. (b) Protein structure of AQP1, FAM188B, and ENST00000434909. (c) Quantitative PCR analysis of ENST00000434909 in normal tissues. ACTB was used for normalization of expression levels. (TIF) [file pgen.1002541.s008.tif]

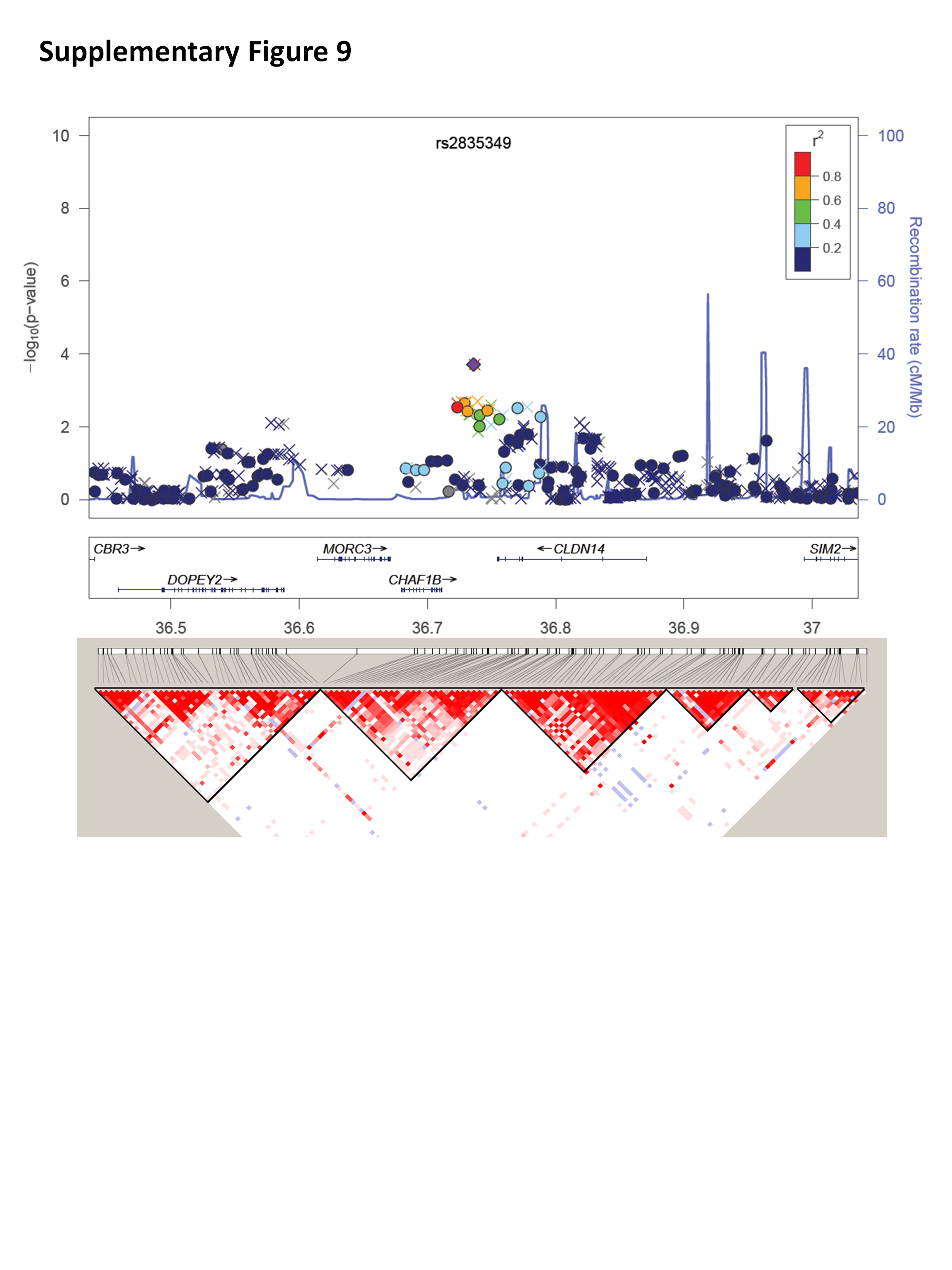

Supplement: Figure S9 — Regional association plots at rs2835349 loci. Upper panel; P-values of genotyped SNPs (circle) and imputed SNPs (cross) are plotted (as −log10 P-value) against their physical position on chromosome 21 (NCBI Build 36). SNPs rs2835349 on 21q22 are represented by purple diamonds. The genetic recombination rates estimated from 1000 Genomes samples (JPT+CHB) are shown with a blue line. SNP's color indicates LD with rs2835349 according to a scale from r 2 = 0 to r 2 = 1 based on pair-wise r 2 values from HapMap JPT. Middle Panel; Gene annotations from the University of California Santa Cruz genome browser. Lower Panel; We drew the LD map based on D' values using the genotype data of the cases and controls in the GWAS samples. (TIF) [file pgen.1002541.s009.tif]
