# Supplementary material for: A Genome-Wide Association Study of Nephrolithiasis in the Japanese Population Identifies Novel Susceptible Loci at 5q35.3, 7p14.3, and 13q14.1
Source: PLoS Genet. 2012 Mar 1;8(3):e1002541. doi: 10.1371/journal.pgen.1002541 (PMC3291538; doi:10.1371/journal.pgen.1002541)
Supplement: Figure S5 — Forest plots for stratified association analysis at rs11746443 (a), rs1000597 (b) and rs4142110 (c). Case and control samples from GWAS, replication 1, and replication 2 were stratified by age (case; ≥60 n = 2006 <60 n = 3689, control; ≥60 n = 8300 <60 n = 8837), gender (case; male n = 4268 female n = 1434, control; male n = 9462 female n = 7863), and BMI (case; ≥24 n = 2592 <24 n = 2656, control; ≥24 n = 4781 <24 n = 10586). Odds ratios and confidence intervals are calculated from fixed effect model implemented in R. The P values of heterogeneities (P het) across three stages examined by using the Breslow-Day test. (PDF) [file pgen.1002541.s005.pdf]

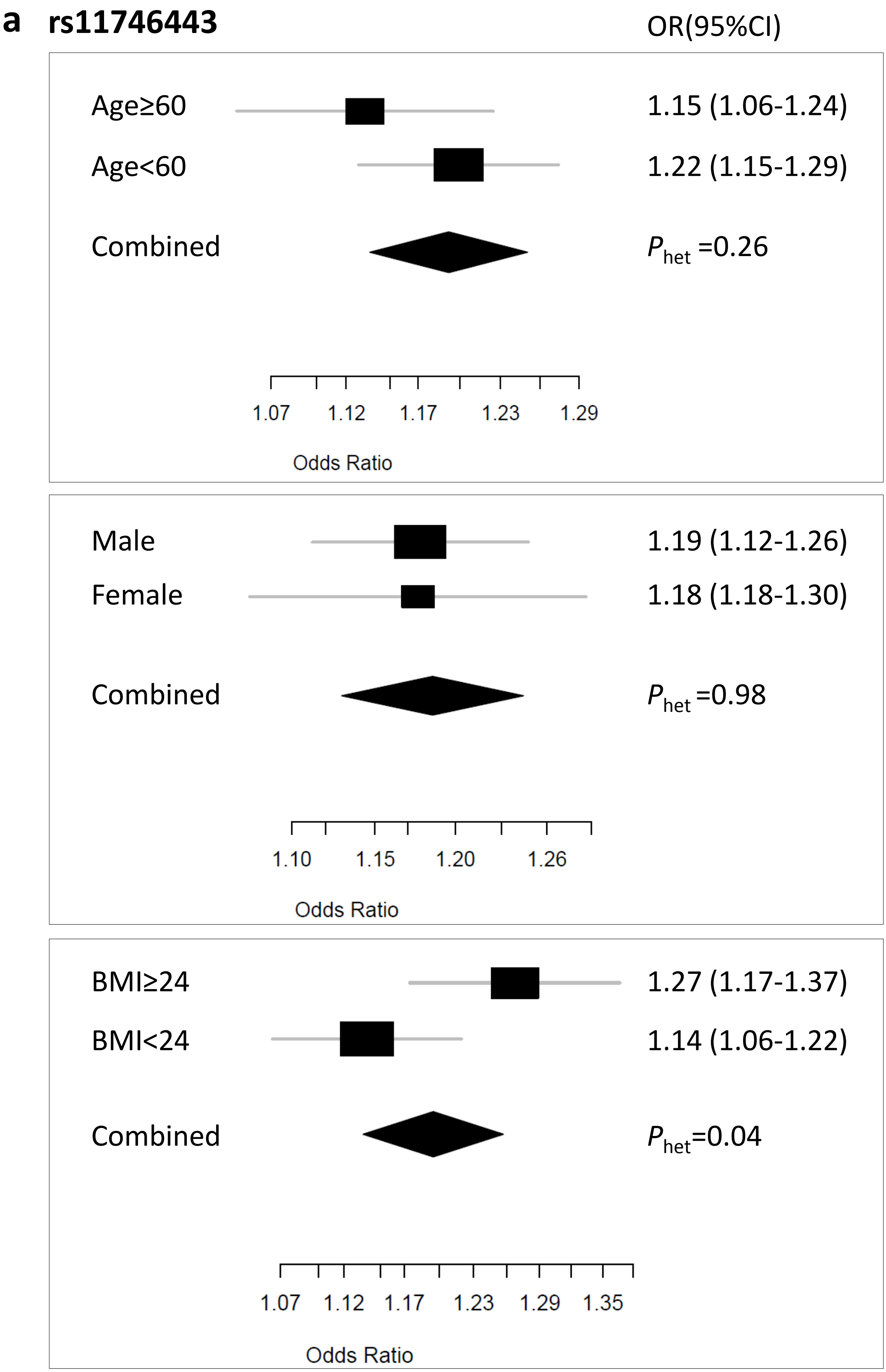
a rs11746443
OR(95%CI)
Age≥60
1.15 (1.06-1.24)
Age<60
1.22 (1.15-1.29)
Combined
P_{het} =0.26
1.07 1.12 1.17 1.23 1.29
Odds Ratio
Male
1.19 (1.12-1.26)
Female
1.18 (1.18-1.30)
Combined
P_{het} =0.98
1.10 1.15 1.20 1.26
Odds Ratio
BMI≥24
1.27 (1.17-1.37)
BMI<24
1.14 (1.06-1.22)
Combined
P_{het}=0.04
1.07 1.12 1.17 1.23 1.29 1.35
Odds Ratio

b **rs1000597**

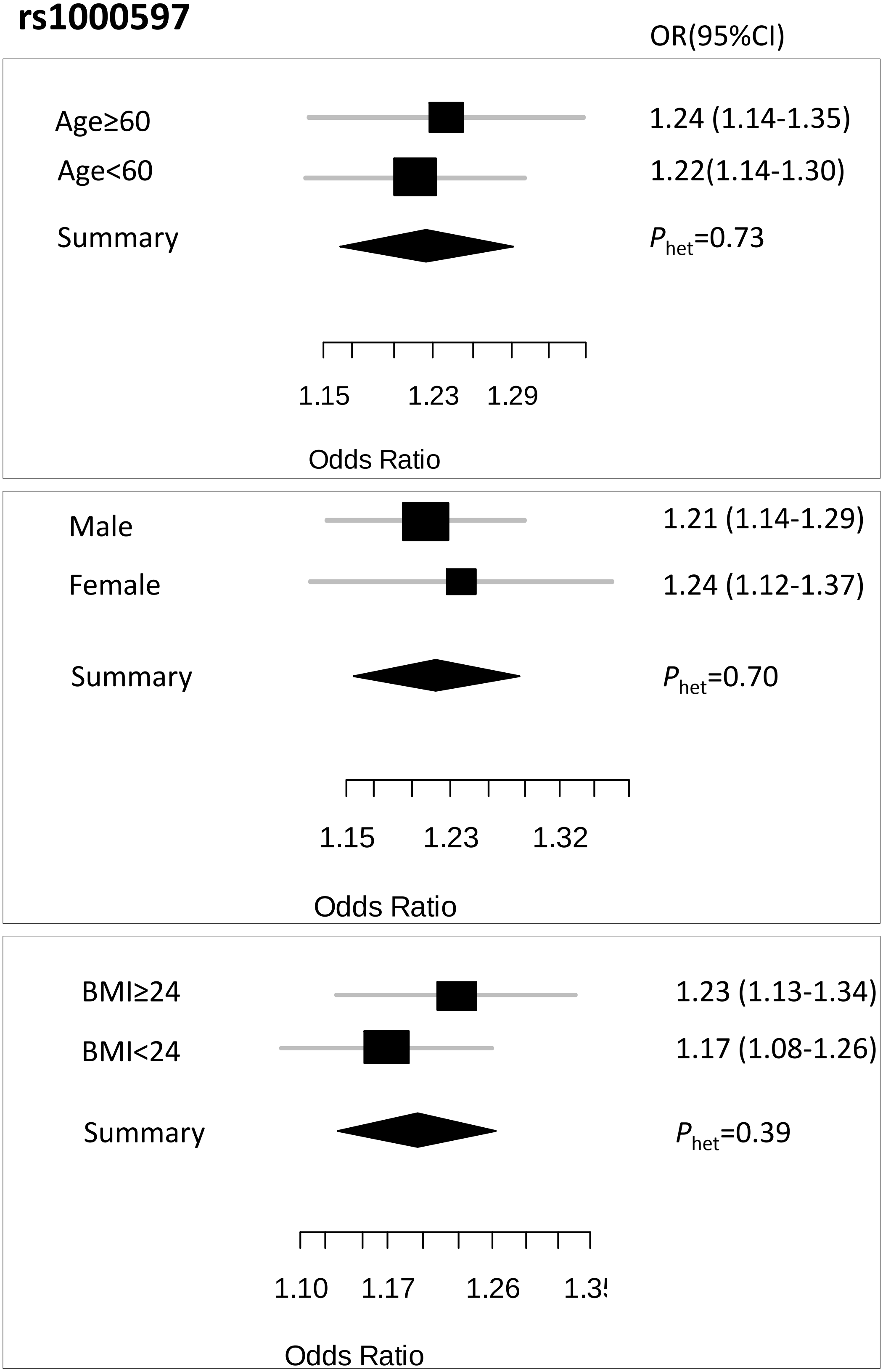
OR(95%CI)
Age≥60
1.24 (1.14-1.35)
Age<60
1.22(1.14-1.30)
Summary
Phet=0.73
1.15
1.23
1.29
Odds Ratio
Male
1.21 (1.14-1.29)
Female
1.24 (1.12-1.37)
Summary
Phet=0.70
1.15
1.23
1.32
Odds Ratio
BMI≥24
1.23 (1.13-1.34)
BMI<24
1.17 (1.08-1.26)
Summary
Phet=0.39
1.10
1.17
1.26
Odds Ratio

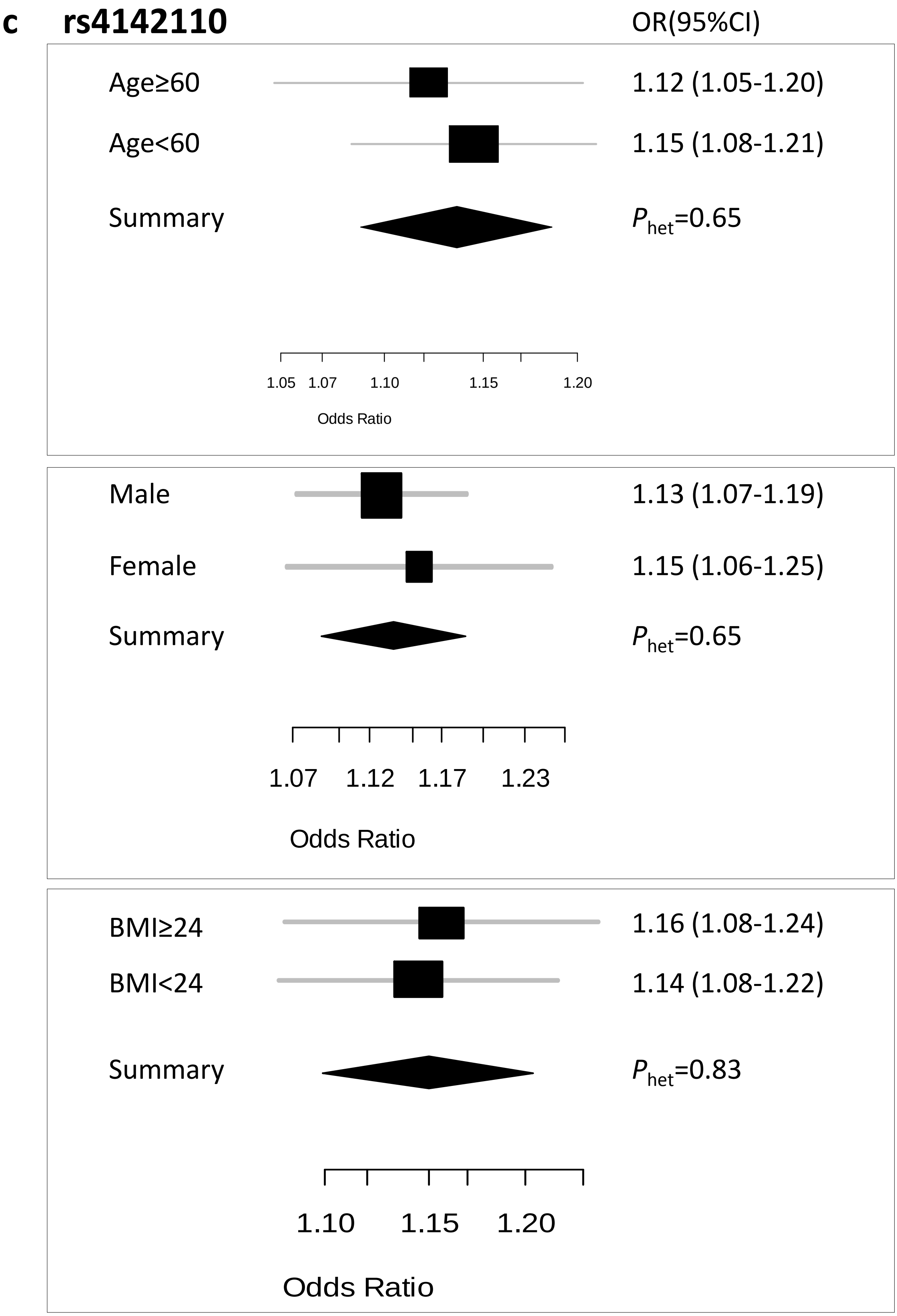
c
rs4142110
OR(95%CI)
Age≥60
1.12 (1.05-1.20)
Age<60
1.15 (1.08-1.21)
Summary
P het=0.65
1.05 1.07 1.10 1.15 1.20
Odds Ratio
Male
1.13 (1.07-1.19)
Female
1.15 (1.06-1.25)
Summary
P het=0.65
1.07 1.12 1.17 1.23
Odds Ratio
BMI≥24
1.16 (1.08-1.24)
BMI<24
1.14 (1.08-1.22)
Summary
P het=0.83
1.10 1.15 1.20
Odds Ratio
