# Supplementary material for: A Genome-Wide Association Study of Nephrolithiasis in the Japanese Population Identifies Novel Susceptible Loci at 5q35.3, 7p14.3, and 13q14.1
Source: PLoS Genet. 2012 Mar 1;8(3):e1002541. doi: 10.1371/journal.pgen.1002541 (PMC3291538; doi:10.1371/journal.pgen.1002541)
Supplement: Table S1 — Characteristics of samples and methods used in this study. (DOCX) [file pgen.1002541.s010.docx]

| **Supplementary Table 1 Characteristics of samples and methods used in this study** | | | | | |
| --- | --- | --- | --- | --- | --- |
| **Stage** | **Source** | **Platform** | **Number of samples** | **Female (%)** | **Age(mean +/- SD)** |
| **GWAS** |  |  |  |  |  |
| Nephrolithiasis | BioBank Japan | Omni Express | 1,000 | 162 (16.2%) | 53.38 +/- 12.23 |
| Control^a,d^ | Healthy volunteer | Omni Express | 7,936 | 3,364 (42.4%) | 58.54 +/- 13.29 |
|  | BioBank Japan |  |  |  |  |
| **Replication1** |  |  |  |  |  |
| Nephrolithiasis | BioBank Japan | Invader assay | 2,783 | 738 (27.3%) | 50.73 +/- 18.07 |
| Control^b,d^ | BioBank Japan | Omni Express | 5,251 | 2,869 (54.6%) | 42.0 +/- 18.40 |
| **Replication2** |  |  |  |  |  |
| Nephrolithiasis | BioBank Japan | Invader assay | 2,109 | 556 (26.6%) | 54.55 +/- 14.68 |
| Control^c,d^ | BioBank Japan | Invader assay | 4,622 | 4,622 (40.5%) | 65.14 +/- 11.10 |
| **QTL analysis** |  |  |  |  |  |
| Samples^e^ | BioBank Japan | Illumina HumanHap 610K | 27,323 | 12,730 (46.5%) | 62.86 +/- 11.98 |
| ^a^Control samples for GWAS contain healthy volunteers, and patients with cerebral aneurysm, primary sclerosing cholangitis, esophageal cancer, uterine body cancer, chronic obstructive pulmonary and glaucoma. ^b^Control samples for replication 1 consist of patients with epilepsy, atrophic dermatitis, and Grave’s disease. ^c^Control samples for replication 2 consist of patients with chronic hepatitis C, liver cancer, and colon cancer. ^d^All subjects with a history of nephrolithiasis were excluded from controls. ^e^All samples for QTL analysis for serum level of phosphorus, calcium and urate, estimate glomerular filtration rate and body mass index consist of patients with Colon cancer, Breast cancer, Prostate cancer, Lung cancer, Gastric cancer, Diabetes, arteriosclerosis obliterans, Atrial fibrillation, Brain infarction, Myocardial infarction, Gall bladder cancer, Pancreas cancer, Anti-cancer drug user, Drug eruption, Rheumatoid arthritis, Warfarin user, amyotrophic lateral sclerosis, Liver cancer, Liver cirrhosis, Uterine fibrosis, and Osteoporosis. | | | | | |
