# Supplementary material for: A Genome-Wide Association Study of Nephrolithiasis in the Japanese Population Identifies Novel Susceptible Loci at 5q35.3, 7p14.3, and 13q14.1
Source: PLoS Genet. 2012 Mar 1;8(3):e1002541. doi: 10.1371/journal.pgen.1002541 (PMC3291538; doi:10.1371/journal.pgen.1002541)
Supplement: Table S2 — The Result of GWAS for nephrolithiasis (Top 100 SNPs). (DOCX) [file pgen.1002541.s011.docx]

| **Supplementary Table 2 The Result of GWAS for nephrolithiasis (Top 100 SNPs) 。.** | | | | | | | | | | | | |
| --- | --- | --- | --- | --- | --- | --- | --- | --- | --- | --- | --- | --- |
| **SNP** | **Chr^a^** | **Position** | ***P*^b^** | **OR^c^** | **95%CI^c^** |  | **SNP** | **Chr^a^** | **Position** | ***P*^b^** | **OR^c^** | **95%CI^c^** |
| rs4838896 | 1 | 111821777 | 5.87x10^-6^ | 1.27 | 1.15-1.41 |  | rs1450997 | 12 | 98029100 | 9.31x10^-7^ | 1.27 | 1.16-1.41 |
| rs12401767 | 1 | 111829797 | 3.36x10^-5^ | 1.25 | 1.12-1.39 |  | rs6538815 | 12 | 98032566 | 3.59x10^-5^ | 1.23 | 1.12-1.36 |
| rs3806446 | 1 | 111832291 | 2.35x10^-5^ | 1.27 | 1.14-1.41 |  | rs34552 | 12 | 98038863 | 2.01x10^-6^ | 1.27 | 1.15-1.40 |
| rs16858527 | 1 | 162187715 | 5.84x10^-5^ | 1.41 | 1.19-1.67 |  | rs10735355 | 12 | 98054607 | 2.19x10^-5^ | 1.23 | 1.12-1.37 |
| rs16858695 | 1 | 162193738 | 5.45x10^-5^ | 1.41 | 1.19-1.67 |  | rs248812 | 12 | 98061146 | 9.38x10^-6^ | 1.25 | 1.14-1.37 |
| rs2279127 | 1 | 203472941 | 5.28x10^-5^ | 1.30 | 1.15-1.48 |  | rs9511023 | 13 | 24631796 | 4.86x10^-5^ | 1.25 | 1.12-1.37 |
| rs916522 | 2 | 12279879 | 6.82x10^-5^ | 1.69 | 1.30-2.20 |  | rs9566906 | 13 | 42642546 | 1.18x10^-5^ | 1.27 | 1.14-1.41 |
| rs2198161 | 2 | 19625636 | 6.86x10^-5^ | 1.38 | 1.18-1.61 |  | rs2253650 | 13 | 42656841 | 7.16x10^-5^ | 1.22 | 1.11-1.35 |
| rs703297 | 2 | 19625818 | 4.11x10^-5^ | 1.38 | 1.18-1.62 |  | rs4994103 | 13 | 42657148 | 1.17x10^-5^ | 1.52 | 1.25-1.82 |
| rs13023094 | 2 | 27910706 | 7.05x10^-5^ | 1.22 | 1.11-1.35 |  | rs1170191 | 13 | 42675493 | 1.16x10^-5^ | 1.25 | 1.12-1.37 |
| rs13405108 | 2 | 139258603 | 6.29x10^-5^ | 1.23 | 1.11-1.37 |  | rs9566921 | 13 | 42687004 | 4.22x10^-6^ | 1.54 | 1.28-1.89 |
| rs861239 | 2 | 170175109 | 4.87x10^-5^ | 1.24 | 1.12-1.38 |  | rs7981733 | 13 | 42690060 | 4.08x10^-7^ | 1.32 | 1.19-1.47 |
| rs6716834 | 2 | 170175334 | 4.51x10^-5^ | 1.24 | 1.12-1.38 |  | rs9566924 | 13 | 42699907 | 2.12x10^-5^ | 1.52 | 1.25-1.82 |
| rs10930352 | 2 | 170196990 | 4.59x10^-5^ | 1.24 | 1.12-1.38 |  | rs1170155 | 13 | 42702711 | 1.15x10^-5^ | 1.27 | 1.14-1.41 |
| rs13015369 | 2 | 234726058 | 4.02x10^-5^ | 1.32 | 1.16-1.52 |  | rs1750017 | 13 | 42704451 | 2.13x10^-5^ | 1.24 | 1.12-1.36 |
| rs11926073 | 3 | 28871595 | 1.74x10^-5^ | 1.46 | 1.23-1.74 |  | rs1170178 | 13 | 42705808 | 1.60x10^-5^ | 1.24 | 1.13-1.37 |
| rs977842 | 5 | 33828203 | 4.62x10^-5^ | 1.28 | 1.14-1.43 |  | rs9533007 | 13 | 42710235 | 1.94x10^-5^ | 1.27 | 1.14-1.41 |
| rs13156926 | 5 | 106116276 | 9.58x10^-6^ | 1.26 | 1.14-1.39 |  | rs4142110 | 13 | 42754522 | 7.15x10^-5^ | 1.22 | 1.11-1.35 |
| rs4075958 | 5 | 176784512 | 3.74x10^-5^ | 1.26 | 1.13-1.40 |  | rs4598803 | 13 | 42762871 | 1.89x10^-5^ | 1.24 | 1.12-1.37 |
| rs12654812 | 5 | 176794191 | 1.98x10^-5^ | 1.24 | 1.13-1.37 |  | rs9566935 | 13 | 42767185 | 1.29x10^-5^ | 1.54 | 1.27-1.85 |
| rs4074995 | 5 | 176797343 | 1.95x10^-5^ | 1.27 | 1.14-1.41 |  | rs1170109 | 13 | 42779694 | 2.50x10^-5^ | 1.23 | 1.12-1.36 |
| rs11746443 | 5 | 176798306 | 1.62x10^-5^ | 1.27 | 1.14-1.41 |  | rs9566939 | 13 | 42782887 | 9.21x10^-6^ | 1.25 | 1.14-1.39 |
| rs10866705 | 5 | 176801131 | 7.43x10^-5^ | 1.23 | 1.11-1.37 |  | rs347413 | 13 | 42787040 | 2.61x10^-5^ | 1.23 | 1.12-1.36 |
| rs7763360 | 6 | 42039572 | 2.21x10^-5^ | 1.40 | 1.20-1.63 |  | rs1990292 | 17 | 59444758 | 2.25x10^-5^ | 1.24 | 1.12-1.37 |
| rs881858 | 6 | 43806609 | 9.90x10^-6^ | 1.36 | 1.19-1.56 |  | rs9905274 | 17 | 59450441 | 2.81x10^-5^ | 1.25 | 1.14-1.39 |
| rs2749083 | 6 | 106836299 | 3.00x10^-5^ | 1.45 | 1.22-1.73 |  | rs3765623 | 18 | 3086065 | 3.83x10^-5^ | 1.38 | 1.18-1.61 |
| rs3765258 | 6 | 136944063 | 7.38x10^-5^ | 1.24 | 1.12-1.38 |  | rs9807753 | 18 | 20664430 | 1.04x10^-5^ | 1.27 | 1.14-1.42 |
| rs1468313 | 7 | 30887782 | 4.19x10^-6^ | 1.31 | 1.16-1.46 |  | rs8098316 | 18 | 20672555 | 4.77x10^-6^ | 1.29 | 1.15-1.43 |
| rs12674155 | 7 | 30899543 | 2.47x10^-6^ | 1.32 | 1.17-1.48 |  | rs6507498 | 18 | 20683093 | 4.55x10^-5^ | 1.24 | 1.12-1.38 |
| rs12669187 | 7 | 30915478 | 1.04x10^-6^ | 1.34 | 1.19-1.51 |  | rs8093542 | 18 | 20709123 | 3.21x10^-6^ | 1.29 | 1.16-1.44 |
| rs4723021 | 7 | 30934659 | 1.61x10^-5^ | 1.29 | 1.15-1.44 |  | rs4800451 | 18 | 20716805 | 3.30x10^-6^ | 1.28 | 1.16-1.43 |
| rs1000597 | 7 | 30937178 | 1.06x10^-5^ | 1.29 | 1.15-1.45 |  | rs4800148 | 18 | 20724328 | 6.35x10^-6^ | 1.28 | 1.15-1.43 |
| rs952368 | 7 | 38046212 | 1.08x10^-5^ | 1.47 | 1.25-1.75 |  | rs7235010 | 18 | 20724810 | 7.59x10^-5^ | 1.26 | 1.12-1.41 |
| rs2040369 | 7 | 142137119 | 5.14x10^-5^ | 1.31 | 1.15-1.50 |  | rs4800452 | 18 | 20727611 | 7.74x10^-5^ | 1.26 | 1.12-1.41 |
| rs7003946 | 8 | 37409288 | 4.73x10^-5^ | 1.23 | 1.11-1.37 |  | rs4239436 | 18 | 20731930 | 4.97x10^-5^ | 1.27 | 1.13-1.43 |
| rs16912805 | 8 | 92406021 | 4.43x10^-5^ | 2.10 | 1.47-3.01 |  | rs4058287 | 18 | 55995721 | 9.29x10^-6^ | 1.26 | 1.14-1.39 |
| rs11251596 | 10 | 2993191 | 2.05x10^-5^ | 1.78 | 1.37-2.33 |  | rs7257356 | 19 | 4013389 | 7.34x10^-5^ | 1.69 | 1.30-2.20 |
| rs2177831 | 10 | 124431063 | 3.76x10^-5^ | 1.33 | 1.16-1.52 |  | rs8113562 | 19 | 4014065 | 5.12x10^-5^ | 1.71 | 1.32-2.22 |
| rs4980250 | 10 | 124456562 | 5.96x10^-5^ | 1.32 | 1.15-1.51 |  | rs3786654 | 19 | 14557821 | 3.30x10^-6^ | 1.30 | 1.16-1.45 |
| rs2901344 | 10 | 124488143 | 6.30x10^-6^ | 1.36 | 1.19-1.55 |  | rs2420538 | 19 | 14573654 | 3.96x10^-6^ | 1.29 | 1.16-1.44 |
| rs17104017 | 10 | 124491040 | 6.69x10^-6^ | 1.36 | 1.19-1.55 |  | rs867174 | 19 | 17952930 | 2.75x10^-6^ | 1.63 | 1.33-2.00 |
| rs13376724 | 10 | 124504595 | 1.57x10^-6^ | 1.38 | 1.21-1.58 |  | rs12327843 | 19 | 18004912 | 2.26x10^-6^ | 1.63 | 1.33-2.00 |
| rs4980182 | 10 | 124506648 | 9.84x10^-6^ | 1.35 | 1.18-1.54 |  | rs13344313 | 19 | 18517767 | 3.21x10^-5^ | 1.27 | 1.14-1.43 |
| rs1445604 | 11 | 105498095 | 1.22x10^-5^ | 1.24 | 1.13-1.37 |  | rs2560966 | 19 | 48531216 | 3.49x10^-5^ | 1.32 | 1.16-1.52 |
| rs10502250 | 11 | 122403463 | 3.63x10^-6^ | 1.31 | 1.17-1.47 |  | rs8105198 | 19 | 48543862 | 2.52x10^-5^ | 1.33 | 1.16-1.52 |
| rs2315027 | 11 | 123770186 | 1.02x10^-6^ | 1.28 | 1.16-1.41 |  | rs6084184 | 20 | 2812772 | 6.67x10^-5^ | 1.25 | 1.12-1.39 |
| rs1732362 | 12 | 62406171 | 5.22x10^-5^ | 1.23 | 1.11-1.37 |  | rs17217119 | 20 | 52742590 | 7.85x10^-5^ | 1.47 | 1.22-1.39 |
| rs11108836 | 12 | 97529985 | 3.68x10^-5^ | 1.41 | 1.20-1.65 |  | rs2835349 | 21 | 37814114 | 6.33x10^-5^ | 1.22 | 1.11-1.35 |
| rs11108857 | 12 | 97553106 | 5.97x10^-5^ | 1.38 | 1.18-1.61 |  | rs2003752 | 22 | 23742105 | 5.89x10^-5^ | 1.27 | 1.14-1.43 |
| rs2160427 | 12 | 97588017 | 2.21x10^-5^ | 1.42 | 1.21-1.68 |  | rs5759802 | 22 | 23779822 | 1.51x10^-5^ | 1.24 | 1.13-1.37 |
| Note: 904 Nephrolithiasis cases and 7,471 controls were analyzed. The result of top 100 SNPs (P < 7.85 x 10^-5^ ) were shown in this Table. ^a^Chr: Chromosome ^b^*P* values obtained from Cochrane-Armitage trend test ^c^Odds ratios (OR) and confidence interval (CI) are calculated using the non-susceptible allele as reference. | | | | | | | | | | | | |
|  | | | | | | | | | | | | |
