# Supplementary material for: A Genome-Wide Association Study of Nephrolithiasis in the Japanese Population Identifies Novel Susceptible Loci at 5q35.3, 7p14.3, and 13q14.1
Source: PLoS Genet. 2012 Mar 1;8(3):e1002541. doi: 10.1371/journal.pgen.1002541 (PMC3291538; doi:10.1371/journal.pgen.1002541)
Supplement: Table S3 — Results of association analysis for nephrolithiasis in stage2 (59 SNPs). (DOCX) [file pgen.1002541.s012.docx]

| **Supplementary Table 3 Results of association analysis for nephrolithiasis in replication 1 (59 SNPs)** | | | | | | | | |
| --- | --- | --- | --- | --- | --- | --- | --- | --- |
| **SNP** | **Chr^a^** | **Position** | **gene** | **Case MAF^b^** | **Control MAF^b^** | ***P*^c^** | **OR^d^** | **95%CI^d^** |
| rs4838896 | 1 | 111821777 | *RP11-165H20.1* | 0.372 | 0.389 | 3.26x10^-2^ | 1.08 | 1.01-1.15 |
| rs16858695 | 1 | 162193738 | *NOS1AP* | 0.124 | 0.121 | 5.38 x10^-1^ | 0.97 | 0.88-1.08 |
| rs2279127 | 1 | 203472941 | *OPTC* | 0.155 | 0.156 | 9.12x10^-1^ | 0.99 | 0.91-1.09 |
| rs916522 | 2 | 12279879 | *LOC100506457* | 0.025 | 0.022 | 2.81x10^-1^ | 1.12 | 0.91-1.39 |
| rs703297 | 2 | 19625818 | *OSR1* | 0.086 | 0.094 | 1.09x10^-1^ | 0.91 | 0.81-1.02 |
| rs13023094 | 2 | 27910706 | *SLC4A1AP* | 0.427 | 0.395 | 5.84x10^-5^ | 1.14 | 1.07-1.22 |
| rs13405108 | 2 | 139258603 | *SPOPL* | 0.350 | 0.373 | 4.37x10^-3^ | 1.11 | 1.03-1.19 |
| rs6716834 | 2 | 170175334 | *LRP2* | 0.296 | 0.295 | 9.70x10^-1^ | 1.00 | 0.93-1.08 |
| rs13015369 | 2 | 234726058 | *HERTR7B1* | 0.125 | 0.127 | 5.95x10^-1^ | 0.97 | 0.88-1.07 |
| rs11926073 | 3 | 28871595 | *No gene* | 0.071 | 0.068 | 5.18x10^-1^ | 1.04 | 0.92-1.18 |
| rs977842 | 5 | 33828203 | *ADAMTS12* | 0.220 | 0.201 | 6.67x10^-3^ | 1.12 | 1.03-1.21 |
| rs13156926 | 5 | 106116276 | *LOC100289569* | 0.367 | 0.361 | 5.02x10^-1^ | 1.02 | 0.96-1.10 |
| rs12654812 | 5 | 176794191 | *RGS14* | 0.381 | 0.354 | 5.57x10^-4^ | 1.13 | 1.05-1.20 |
| rs11746443 | 5 | 176798306 | *RGS14* | 0.283 | 0.252 | 1.93x10^-5^ | 1.17 | 1.09-1.26 |
| rs10866705 | 5 | 176801131 | No gene | 0.359 | 0.306 | 4.62x10^-11^ | 1.27 | 1.18-1.36 |
| rs7763360 | 6 | 42039572 | *TAF8* | 0.097 | 0.094 | 5.97x10^-1^ | 1.03 | 0.92-1.15 |
| rs881858 | 6 | 43806609 | *LOC100132354* | 0.130 | 0.117 | 1.73x10^-2^ | 1.13 | 1.02-1.24 |
| rs2749083 | 6 | 106836299 | *LOC100506165* | 0.065 | 0.068 | 6.07x10^-1^ | 0.97 | 0.85-1.10 |
| rs3765258 | 6 | 136944063 | *MAP3K5* | 0.269 | 0.272 | 6.39x10^-1^ | 0.98 | 0.91-1.06 |
| rs12669187 | 7 | 30915478 | *FAM188B* | 0.214 | 0.184 | 4.56x10^-6^ | 1.21 | 1.11-1.31 |
| rs1000597 | 7 | 30937178 | No gene | 0.241 | 0.204 | 4.60x10^-8^ | 1.24 | 1.15-1.34 |
| rs952368 | 7 | 38046212 | *EPDR1* | 0.112 | 0.105 | 2.03x10^-1^ | 0.93 | 0.84-1.04 |
| rs2040369 | 7 | 142137119 | *TRB@* | 0.133 | 0.132 | 8.13x10^-1^ | 1.01 | 0.92-1.11 |
| rs7003946 | 8 | 37409288 | No gene | 0.388 | 0.407 | 1.79x10^-2^ | 1.08 | 1.01-1.16 |
| rs16912805 | 8 | 92406021 | *SLC26A7* | 0.014 | 0.013 | 6.05x10^-1^ | 1.08 | 0.81-1.42 |
| rs11251596 | 10 | 2993191 | *PFKP* | 0.023 | 0.022 | 8.25x10^-1^ | 1.02 | 0.82-1.28 |
| rs2177831 | 10 | 124431063 | No gene | 0.147 | 0.135 | 4.30x10^-2^ | 1.10 | 1.00-1.21 |
| rs13376724 | 10 | 124504595 | *FLJ46361* | 0.149 | 0.143 | 3.24x10^-1^ | 1.05 | 0.96-1.15 |
| rs1445604 | 11 | 105498095 | *GRIA4* | 0.482 | 0.473 | 2.71x10^-1^ | 1.04 | 0.97-1.11 |
| rs2315027 | 11 | 123770186 | *OR8D4* | 0.402 | 0.398 | 6.41x10^-1^ | 1.02 | 0.95-1.09 |
| rs2160427 | 12 | 97588017 | No gene | 0.079 | 0.071 | 9.79x10^-2^ | 1.11 | 0.98-1.25 |
| rs1450997 | 12 | 98029100 | *MIR135A2* | 0.468 | 0.481 | 1.03x10^-1^ | 0.95 | 0.89-1.01 |
| rs6538815 | 12 | 98032566 | No gene | 0.346 | 0.357 | 2.02x10^-1^ | 0.96 | 0.89-1.02 |
| rs248812 | 12 | 98061146 | *LOC643711* | 0.469 | 0.464 | 6.24x10^-1^ | 0.98 | 0.92-1.05 |
| rs9511023 | 13 | 24631796 | No gene | 0.318 | 0.324 | 4.02x10^-1^ | 1.03 | 0.96-1.10 |
| rs2253650 | 13 | 42656841 | *DGKH* | 0.471 | 0.495 | 3.91x10^-3^ | 1.10 | 1.03-1.18 |
| rs4994103 | 13 | 42657148 | *DGKH* | 0.098 | 0.105 | 2.19x10^-1^ | 1.08 | 0.96-1.19 |
| rs9566921 | 13 | 42687004 | *DGKH* | 0.092 | 0.101 | 7.47x10^-2^ | 1.11 | 0.99-1.23 |
| rs7981733 | 13 | 42690060 | *DGKH* | 0.314 | 0.341 | 5.09x10^-4^ | 1.14 | 1.05-1.22 |
| rs1170155 | 13 | 42702711 | *DGKH* | 0.328 | 0.364 | 7.01x10^-6^ | 1.18 | 1.10-1.25 |
| rs1170178 | 13 | 42705808 | *DGKH* | 0.466 | 0.437 | 4.54x10^-4^ | 1.12 | 1.05-1.20 |
| rs4142110 | 13 | 42754522 | *DGKH* | 0.430 | 0.458 | 7.03x10^-4^ | 1.12 | 1.05-1.19 |
| rs4598803 | 13 | 42762871 | *DGKH* | 0.441 | 0.414 | 1.19x10^-3^ | 1.11 | 1.04-1.19 |
| rs1990292 | 17 | 59444758 | *BCAS3* | 0.395 | 0.376 | 1.57x10^-2^ | 1.09 | 1.02-1.16 |
| rs9905274 | 17 | 59450441 | *BCAS3* | 0.480 | 0.493 | 1.12x10^-1^ | 1.05 | 0.99-1.12 |
| rs3765623 | 18 | 3086065 | *MYOM1* | 0.108 | 0.091 | 7.15x10^-4^ | 1.21 | 1.08-1.34 |
| rs6507498 | 18 | 20683093 | No gene | 0.293 | 0.294 | 8.89x10^-1^ | 0.99 | 0.93-1.07 |
| rs8093542 | 18 | 20709123 | *CABLES1* | 0.251 | 0.249 | 8.69x10^-1^ | 1.01 | 0.93-1.08 |
| rs4800148 | 18 | 20724328 | *CABLES1* | 0.254 | 0.255 | 8.44x10^-1^ | 0.99 | 0.92-1.07 |
| rs4058287 | 18 | 55995721 | *NEDD4L* | 0.338 | 0.341 | 7.33x10^-1^ | 0.99 | 0.92-1.06 |
| rs8113562 | 19 | 4014065 | *PIAS4* | 0.034 | 0.027 | 1.82x10^-2^ | 1.25 | 1.04-1.51 |
| rs3786654 | 19 | 14557821 | *PKN1* | 0.253 | 0.236 | 1.80x10^-2^ | 1.10 | 1.02-1.18 |
| rs12327843 | 19 | 18004912 | *SLC5A5* | 0.042 | 0.044 | 7.01x10^-1^ | 0.97 | 0.82-1.14 |
| rs13344313 | 19 | 18517767 | No gene | 0.217 | 0.218 | 9.50x10^-1^ | 1.00 | 0.92-1.08 |
| rs8105198 | 19 | 48543862 | *CABP5* | 0.184 | 0.193 | 1.49x10^-1^ | 1.02 | 0.98-1.15 |
| rs6084184 | 20 | 2812772 | *FAM133A* | 0.290 | 0.313 | 2.45x10^-3^ | 1.11 | 1.04-1.20 |
| rs17217119 | 20 | 52742590 | *CYP24A1* | 0.084 | 0.094 | 3.79x10^-2^ | 1.12 | 1.01-1.27 |
| rs2835349 | 21 | 37814114 | *CLDN14* | 0.482 | 0.506 | 3.29x10^-3^ | 1.10 | 1.03-1.18 |
| rs2003752 | 22 | 23742105 | No gene | 0.256 | 0.262 | 3.96x10^-1^ | 1.03 | 0.96-1.11 |
| Note: 2,783 Nephrolithiasis cases and 5,251 controls were analyzed. ^a^Chr: chromosome  ^b^MAF: minor allele frequency ^c^*P* value obtained from Cochrane-Armitage trend test. ^d^Odds ratios (OR) and confidence interval (CI) are calculated using the non-susceptible allele as reference. | | | | | | | | |
