# Supplementary material for: A Genome-Wide Association Study of Nephrolithiasis in the Japanese Population Identifies Novel Susceptible Loci at 5q35.3, 7p14.3, and 13q14.1
Source: PLoS Genet. 2012 Mar 1;8(3):e1002541. doi: 10.1371/journal.pgen.1002541 (PMC3291538; doi:10.1371/journal.pgen.1002541)
Supplement: Table S4 — Results of meta-analysis between GWAS and Stage 2 for nephrolithiasis (59 SNPs). (DOCX) [file pgen.1002541.s013.docx]

| **Supplementary Table 4 Meta-analysis of GWAS and Stage 2 (59 SNPs)** | | | | | | | |
| --- | --- | --- | --- | --- | --- | --- | --- |
| Chr^a^ | SNP | position | gene | *P*^b^ | OR^c^ | 95%CI^c^ | P_het_^d^ |
| 1 | rs4838896 | 111821777 | *RP11-165H20.1* | 1.68x10^-5^ | 1.13 | (1.07-1.20) | 7.73x10^-3^ |
| 1 | rs16858695 | 162193738 | *NOS1AP* | 0.091 | 1.08 | (0.99-1.17) | 1.63x10^-4^ |
| 1 | rs2279127 | 203472941 | *OPTC* | 0.035 | 0.92 | (0.86-0.99) | 7.34x10^-4^ |
| 2 | rs916522 | 12279879 | *LOC100506457* | 2.03x10^-3^ | 1.30 | (1.10-1.54) | 0.017 |
| 2 | rs703297 | 19625818 | *OSR1* | 0.365 | 1.04 | (0.95-1.15) | 2.09x10^-5^ |
| 2 | rs13023094 | 27910706 | *SLC4A1AP* | 2.89x10^-8^ | 1.17 | (1.11-1.23) | 0.265 |
| 2 | rs13405108 | 139258603 | *SPOPL* | 4.21x10^-6^ | 1.14 | (1.08-1.21) | 0.077 |
| 2 | rs6716834 | 170175334 | *LRP2* | 0.022 | 1.07 | (1.01-1.14) | 7.69x10^-4^ |
| 2 | rs13015369 | 234726058 | *HERTR7B1* | 0.074 | 1.08 | (0.99-1.16) | 2.87x10^-4^ |
| 3 | rs11926073 | 28871595 | *No gene* | 3.83x10^-3^ | 1.16 | (1.05-1.29) | 1.99x10^-3^ |
| 5 | rs977842 | 33828203 | *ADAMTS12* | 6.01x10^-6^ | 1.16 | (1.09-1.24) | 0.063 |
| 5 | rs13156926 | 106116276 | *LOC100289569* | 2.61x10^-3^ | 1.09 | (1.03-1.15) | 9.21x10^-4^ |
| 5 | rs12654812 | 176794191 | *RGS14* | 1.78x10^-7^ | 1.16 | (1.10-1.23) | 0.105 |
| 5 | rs11746443 | 176798306 | *RGS14* | 3.06x10^-9^ | 1.20 | (1.13-1.28) | 0.232 |
| 5 | rs10866705 | 176801131 | *No gene* | 5.83x10^-15^ | 1.26 | (1.19-1.33) | 0.661 |
| 6 | rs7763360 | 42039572 | *TAF8* | 5.78x10^-3^ | 1.14 | (1.04-1.24) | 1.58x10^-3^ |
| 6 | rs881858 | 43806609 | *LOC100132354* | 1.01x10^-5^ | 1.20 | (1.11-1.30) | 0.029 |
| 6 | rs2749083 | 106836299 | *LOC100506165* | 0.070 | 1.10 | (0.99-1.23) | 2.87x10^-4^ |
| 6 | rs3765258 | 136944063 | *MAP3K5* | 0.072 | 1.06 | (1.00-1.12) | 3.98x10^-4^ |
| 7 | rs12669187 | 30915478 | *FAM188B* | 1.04x10^-10^ | 1.25 | (1.17-1.33) | 0.150 |
| 7 | rs1000597 | 30937178 | *No gene* | 4.23x10^-12^ | 1.26 | (1.18-1.34) | 0.567 |
| 7 | rs952368 | 38046212 | *EPDR1* | 0.153 | 1.07 | (0.98-1.17) | 8.76x10^-6^ |
| 7 | rs2040369 | 142137119 | *TRB@* | 0.014 | 1.10 | (1.02-1.19) | 1.74x10^-3^ |
| 8 | rs7003946 | 37409288 | *No gene* | 2.57x10^-5^ | 1.13 | (1.07-1.19) | 0.039 |
| 8 | rs16912805 | 92406021 | *SLC26A7* | 7.72x10^-5^ | 1.17 | (1.08-1.26) | 0.024 |
| 10 | rs11251596 | 2993191 | *PFKP* | 5.67x10^-4^ | 1.14 | (1.06-1.23) | 7.52x10^-4^ |
| 10 | rs2177831 | 124431063 | *No gene* | 7.72x10^-5^ | 1.17 | (1.08-1.26) | 0.024 |
| 10 | rs13376724 | 124504595 | *FLJ46361* | 5.67x10^-4^ | 1.14 | (1.06-1.23) | 7.52x10^-4^ |
| 11 | rs1445604 | 105498095 | *GRIA4* | 8.46x10^-4^ | 0.91 | (0.86-0.96) | 2.29x10^-3^ |
| 11 | rs2315027 | 123770186 | *OR8D4* | 1.75x10^-3^ | 1.09 | (1.03-1.15) | 1.26x10^-4^ |
| 12 | rs2160427 | 97588017 | *No gene* | 1.82x10^-4^ | 1.21 | (1.09-1.33) | 0.017 |
| 12 | rs1450997 | 98029100 | *MIR135A2* | 0.186 | 1.04 | (0.98-1.10) | 6.87x10^-7^ |
| 12 | rs6538815 | 98032566 | *No gene* | 0.234 | 1.04 | (0.98-1.10) | 3.97x10^-5^ |
| 12 | rs248812 | 98061146 | *LOC643711* | 0.043 | 1.06 | (1.00-1.12) | 7.57x10^-5^ |
| 13 | rs9511023 | 24631796 | *No gene* | 2.87x10^-3^ | 1.09 | (1.03-1.16) | 2.89x10^-3^ |
| 13 | rs2253650 | 42656841 | *DGKH* | 4.46x10^-6^ | 1.14 | (1.08-1.20) | 0.084 |
| 13 | rs4994103 | 42657148 | *DGKH* | 6.26x10^-4^ | 1.18 | (1.07-1.29) | 1.63x10^-3^ |
| 13 | rs9566921 | 42687004 | *DGKH* | 6.42x10^-5^ | 1.22 | (1.11-1.34) | 2.50x10^-3^ |
| 13 | rs7981733 | 42690060 | *DGKH* | 1.08x10^-8^ | 1.19 | (1.12-1.26) | 0.019 |
| 13 | rs1170155 | 42702711 | *DGKH* | 5.11x10^-10^ | 1.20 | (1.13-1.27) | 0.241 |
| 13 | rs1170178 | 42705808 | *DGKH* | 1.15x10^-7^ | 1.16 | (1.10-1.22) | 0.099 |
| 13 | rs4142110 | 42754522 | *DGKH* | 5.13x10^-7^ | 1.15 | (1.09-1.22) | 0.147 |
| 13 | rs4598803 | 42762871 | *DGKH* | 4.20x10^-7^ | 1.15 | (1.09-1.22) | 0.081 |
| 17 | rs1990292 | 59444758 | *BCAS3* | 1.33x10^-5^ | 1.13 | (1.07-1.20) | 0.033 |
| 17 | rs9905274 | 59450441 | *BCAS3* | 2.56x10^-4^ | 1.11 | (1.05-1.17) | 9.09x10^-3^ |
| 18 | rs3765623 | 3086065 | *MYOM1* | 4.35x10^-7^ | 1.26 | (1.15-1.37) | 0.158 |
| 18 | rs6507498 | 20683093 | *No gene* | 0.034 | 1.07 | (1.01-1.13) | 6.08x10^-4^ |
| 18 | rs8093542 | 20709123 | *CABLES1* | 6.96x10^-3^ | 1.09 | (1.02-1.16) | 1.98x10^-4^ |
| 18 | rs4800148 | 20724328 | *CABLES1* | 0.020 | 1.08 | (1.01-1.14) | 1.30x10^-4^ |
| 18 | rs4058287 | 55995721 | *NEDD4L* | 0.031 | 1.06 | (1.01-1.13) | 1.10x10^-4^ |
| 19 | rs8113562 | 4014065 | *PIAS4* | 4.34x10^-5^ | 1.38 | (1.18-1.60) | 0.055 |
| 19 | rs3786654 | 14557821 | *PKN1* | 4.8x10^-6^ | 1.16 | (1.09-1.23) | 0.012 |
| 19 | rs12327843 | 18004912 | *SLC5A5* | 0.023 | 1.16 | (1.02-1.32) | 7.99x10^-5^ |
| 19 | rs13344313 | 18517767 | *No gene* | 0.026 | 1.08 | (1.01-1.15) | 5.37x10^-4^ |
| 19 | rs8105198 | 48543862 | *CABP5* | 4.26x10^-4^ | 1.14 | (1.06-1.22) | 5.51x10^-3^ |
| 20 | rs6084184 | 2812772 | *FAM133A* | 2.23x10^-6^ | 1.16 | (1.09-1.23) | 0.093 |
| 20 | rs17217119 | 52742590 | *CYP24A1* | 1.04x10^-4^ | 1.22 | (1.10-1.35) | 0.021 |
| 21 | rs2835349 | 37814114 | *CLDN14* | 3.21x10^-6^ | 1.14 | (1.08-1.20) | 0.089 |
| 22 | rs2003752 | 23742105 | *No gene* | 3.53x10^-3^ | 1.10 | (1.03-1.17) | 3.32x10^-3^ |
| Note: 3687 Nephrolithiasis cases (904 samples in GWAS and 2,783 in Stage2) and 12,722 controls (7,471 in GWAS and 5,251 in Stage2) were analyzed. Odds ratio and *P* value for independence test were calculated by Mendel-hauzen and Laird method in the Meta-analysis. ^a^Chr: chromosome  ^b^*P* value obtained from Cochrane-Armitage trend test ^c^Odds ratios (OR) and confidence interval (CI) are calculated using the non-susceptible allele as reference. ^d^The *P* values of heterogeneities (*P*_het_) across three stages examined by using the Breslow-Day test | | | | | | | |
