# Supplementary material for: A Genome-Wide Association Study of Nephrolithiasis in the Japanese Population Identifies Novel Susceptible Loci at 5q35.3, 7p14.3, and 13q14.1
Source: PLoS Genet. 2012 Mar 1;8(3):e1002541. doi: 10.1371/journal.pgen.1002541 (PMC3291538; doi:10.1371/journal.pgen.1002541)
Supplement: Table S5 — a) Results of association analysis for nephrolithiasis in stage3/replication (11 SNPs). b) Results of meta analysis of 3 stage (11SNPs). (DOCX) [file pgen.1002541.s014.docx]

| **Supplementary Table 5a Results of association analysis for nephrolithiasis in stage3/replication** | | | | | | | | |
| --- | --- | --- | --- | --- | --- | --- | --- | --- |
| **SNP** | **Chr^a^** | **Position** | **gene** | **Case MAF^b^** | **Control MAF^b^** | ***P*^c^** | **OR^d^** | **95%CI^d^** |
| rs13023094 | 2 | 27910706 | *SLC4A1AP* | 0.410 | 0.400 | 2.84x10^-1^ | 1.04 | 0.97-1.12 |
| rs12654812 | 5 | 176794191 | *RGS14* | 0.386 | 0.350 | 5.27x10^-5^ | 1.17 | 1.08-1.26 |
| rs11746443 | 5 | 176798306 | *RGS14* | 0.278 | 0.250 | 4.64x10^-4^ | 1.16 | 1.07-1.26 |
| rs12669187 | 7 | 30915478 | *FAM188B* | 0.205 | 0.182 | 1.59x10^-3^ | 1.16 | 1.06-1.27 |
| rs1000597 | 7 | 30937178 | No gene | 0.230 | 0.203 | 5.43x10^-4^ | 1.17 | 1.07-1.28 |
| rs7981733 | 13 | 42690060 | *DGKH* | 0.318 | 0.334 | 7.05x10^-2^ | 1.08 | 0.99-1.16 |
| rs1170155 | 13 | 42702711 | *DGKH* | 0.339 | 0.353 | 1.32x10^-1^ | 1.06 | 0.98-1.15 |
| rs1170178 | 13 | 42705808 | *DGKH* | 0.465 | 0.450 | 1.11x10^-1^ | 1.06 | 0.99-1.14 |
| rs4142110 | 13 | 42754522 | *DGKH* | 0.431 | 0.459 | 2.18x10^-3^ | 1.12 | 1.04-1.20 |
| rs3765623 | 18 | 3086065 | *MYOM1* | 0.109 | 0.098 | 3.71x10^-2^ | 1.13 | 1.01-1.28 |
| Note: 2,109 Nephrolithiasis cases and 4,622 controls were analyzed. ^a^Chr: Chromosome. ^b^MAF: minor allele frequency. ^c^*P* value obtained from Cochrane-Armitage trend test. ^d^Odds ratios (OR) and confidence interval (CI) are calculated using the non-susceptible allele as reference. | | | | | | | | |

| **Supplementary Table 5b Results of meta analysis of 3stages** | | | | | | | | | | |
| --- | --- | --- | --- | --- | --- | --- | --- | --- | --- | --- |
| Chr^a^ | SNP | stage | allele | gene | Case MAF^b^ | Control MAF^b^ | *P*^c^ | OR^d^ | 95%CI^d^ | P_het_^e^ |
| 2 | rs13023094 | GWAS | C/A | *SLC4A1AP* (2p23.2) | 0.438 | 0.389 | 7.05x10^-5^ | 1.22 | (1.11-1.35) |  |
|  |  | Screening2 |  |  | 0.427 | 0.395 | 5.84x10^-5^ | 1.14 | (1.07-1.22) |  |
|  |  | Screening3 |  |  | 0.410 | 0.400 | 2.84x10^-1^ | 1.04 | (0.97-1.12) |  |
|  |  | Combined^e^ |  |  |  |  | 3.61x10^-7^ | 1.12 | (1.07-1.17) | 0.027 |
| 5 | rs12654812 | GWAS | T/C | *RGS14* (5q35.3) | 0.397 | 0.346 | 1.98x10^-5^ | 1.24 | (1.13-1.37) |  |
|  |  | Screening2 |  |  | 0.381 | 0.354 | 1.34x10^-7^ | 1.14 | (1.09-1.2) |  |
|  |  | Screening3 |  |  | 0.386 | 0.350 | 5.27x10^-5^ | 1.17 | (1.11-1.23) |  |
|  |  | Combined^e^ |  |  |  |  | 4.42x10^-11^ | 1.16 | (1.11-1.22) | 0.267 |
| 5 | rs11746443 | GWAS | T/C | *RGS14* (5q35.3) | 0.290 | 0.244 | 1.62x10^-5^ | 1.27 | (1.14-1.41) |  |
|  |  | Screening2 |  |  | 0.283 | 0.252 | 3.00x10^-8^ | 1.17 | (1.1-1.23) |  |
|  |  | Screening3 |  |  | 0.278 | 0.250 | 4.64x10^-4^ | 1.16 | (1.07-1.26) |  |
|  |  | Combined^e^ |  |  |  |  | 8.51x10^-12^ | 1.19 | (1.13-1.24) | 0.378 |
| 7 | rs12669187 | GWAS | T/C | *FAM188B* (7p14.3) | 0.223 | 0.176 | 1.04x10^-6^ | 1.34 | (1.19-1.51) |  |
|  |  | Screening2 |  |  | 0.214 | 0.184 | 2.70x10^-8^ | 1.19 | (1.12-1.26) |  |
|  |  | Screening3 |  |  | 0.205 | 0.182 | 1.59x10^-3^ | 1.16 | (1.06-1.27) |  |
|  |  | Combined^e^ |  |  |  |  | 1.48x10^-12^ | 1.25 | (1.17-1.33) | 0.150 |
| 7 | rs1000597 | GWAS | G/A | No gene (7p14.3) | 0.247 | 0.202 | 1.06x10^-5^ | 1.29 | (1.15-1.45) |  |
|  |  | Screening2 |  |  | 0.241 | 0.204 | 1.42x10^-10^ | 1.21 | (1.14-1.28) |  |
|  |  | Screening3 |  |  | 0.230 | 0.203 | 5.43x10^-4^ | 1.17 | (1.07-1.28) |  |
|  |  | Combined^e^ |  |  |  |  | 2.16x10^-14^ | 1.22 | (1.15-1.28) | 0.370 |
| 13 | rs7981733 | GWAS | A/G | *DGKH* (13q14.1) | 0.283 | 0.342 | 4.08x10^-7^ | 1.32 | (1.19-1.47) |  |
|  |  | Screening2 |  |  | 0.314 | 0.341 | 1.54x10^-4^ | 1.11 | (1.05-1.16) |  |
|  |  | Screening3 |  |  | 0.318 | 0.334 | 7.05x10^-2^ | 1.08 | (0.99-1.16) |  |
|  |  | Combined^e^ |  |  |  |  | 1.43x10^-8^ | 1.14 | (1.09-1.20) | 0.009 |
| 13 | rs1170155 | GWAS | A/G | *DGKH* (13q14.1) | 0.306 | 0.359 | 1.15x10^-5^ | 1.27 | (1.14-1.41) |  |
|  |  | Screening2 |  |  | 0.328 | 0.364 | 1.32x10^-5^ | 1.12 | (1.07-1.18) |  |
|  |  | Screening3 |  |  | 0.339 | 0.353 | 1.32x10^-1^ | 1.06 | (0.98-1.15) |  |
|  |  | Combined^e^ |  |  |  |  | 3.89x10^-9^ | 1.15 | (1.10-1.20) | 0.021 |
| 13 | rs1170178 | GWAS | A/G | *DGKH* (13q14.1) | 0.494 | 0.440 | 1.60x10^-5^ | 1.24 | (1.13-1.37) |  |
|  |  | Screening2 |  |  | 0.466 | 0.437 | 2.91x10^-4^ | 1.09 | (1.04-1.15) |  |
|  |  | Screening3 |  |  | 0.465 | 0.450 | 1.11x10^-1^ | 1.06 | (0.99-1.14) |  |
|  |  | Combined^e^ |  |  |  |  | 1.91x10^-7^ | 1.12 | (1.08-1.17) | 0.043 |
| 13 | rs4142110 | GWAS | A/G | *DGKH* (13q14.1) | 0.410 | 0.460 | 7.15x10^-5^ | 1.22 | (1.11-1.35) |  |
|  |  | Screening2 |  |  | 0.430 | 0.458 | 4.72x10^-6^ | 1.12 | (1.07-1.18) |  |
|  |  | Screening3 |  |  | 0.431 | 0.459 | 2.18x10^-3^ | 1.12 | (1.04-1.20) |  |
|  |  | Combined^e^ |  |  |  |  | 4.62x10^-9^ | 1.14 | (1.09-1.19) | 0.301 |
| 18 | rs3765623 | GWAS | A/G | *MYOM1* (18p11.3) | 0.115 | 0.086 | 3.82x10^-5^ | 1.38 | (1.18-1.61) |  |
|  |  | Screening2 |  |  | 0.108 | 0.091 | 1.13x10^-4^ | 1.17 | (1.08-1.27) |  |
|  |  | Screening3 |  |  | 0.109 | 0.098 | 3.71x10^-2^ | 1.13 | (1.01-1.28) |  |
|  |  | Combined^e^ |  |  |  |  | 1.28x10^-7^ | 1.21 | (1.13-1.30) | 0.141 |
| Note: 5,796 (904 in GWAS, 2,783 in stage2 and 2,109 in stage3) Nephrolithiasis cases and 17,344 (7,471 in GWAS, 5251 in stage2 and 4,622 in stage3) controls were analyzed.  ^a^Chr: chromosome  ^b^MAF: minor allele frequency ^c^*P* value obtained from Cochrane-Armitage trend test. ^d^Odds ratios (OR) and confidence interval (CI) are calculated using the non-susceptible allele as reference. ^e^The *P* values of heterogeneities (*P*_het_) across three stages examined by using the Breslow-Day test ^f^Meta-analysis: Odds ratio and *P* value for independence test were calculated by Mantel-haenszel test in the Meta-analysis. | | | | | | | | | | |
