# Supplementary material for: A Genome-Wide Association Study of Nephrolithiasis in the Japanese Population Identifies Novel Susceptible Loci at 5q35.3, 7p14.3, and 13q14.1
Source: PLoS Genet. 2012 Mar 1;8(3):e1002541. doi: 10.1371/journal.pgen.1002541 (PMC3291538; doi:10.1371/journal.pgen.1002541)
Supplement: Table S6 — The results of conditional analysis for significantly SNPs in 5q35.3 or 7p14.3 associated with nephrolithiasis in all stages. (DOCX) [file pgen.1002541.s015.docx]

| **Supplementary Table 6. The results of conditional analyses on significant SNPs at 5q35.3 or 7p14.3**  5q35.3 | | | | | |
| --- | --- | --- | --- | --- | --- |
| SNP | *P*^a^ | *P* _adjusted for rs12654812_ | OR(95% CI) | *P* _adjusted for rs11746443_ | OR(95% CI) |
| rs12654812 | 4.42x10^-11^ | NA | - | 0.037 | 1.08(1.00-1.15) |
| rs11746443 | 8.52x10^-12^ | 2.90 x 10^-3^ | 1.12(1.04-1.21) | NA | - |
| 7p14.3 |  |  |  |  |  |
| SNP | *P*^a^ | *P* _adjusted for rs12669187_ | OR(95% CI) | *P* _adjusted for rs1000597_ | OR(95% CI) |
| rs12669187 | 1.48x10^-12^ | NA | - | 0.041 | 1.10(1.00-1.20) |
| rs1000597 | 2.16x10^-14^ | 2.29 x 10^-3^ | 1.14(1.05-1.24) | NA | - |
| Trend *P* are shown with or without adjusting the analysis for the significantly associated SNPs in 5q35.3 or 7p14.3.  ^a^ Meta-analysis *P* was calculated by Mantel-Haenszel test. | | | | | |
