# Supplementary material for: A Genome-Wide Association Study of Nephrolithiasis in the Japanese Population Identifies Novel Susceptible Loci at 5q35.3, 7p14.3, and 13q14.1
Source: PLoS Genet. 2012 Mar 1;8(3):e1002541. doi: 10.1371/journal.pgen.1002541 (PMC3291538; doi:10.1371/journal.pgen.1002541)
Supplement: Table S7 — Result of imputation analysis at 5q35.3. (DOCX) [file pgen.1002541.s016.docx]

| **Supplementary Table 7 Result of imputation analysis at 5q35.3** | | | | | | |
| --- | --- | --- | --- | --- | --- | --- |
| SNP | Position | *P^a^* | rs11746443 | |  | status |
|  |  |  | *D* | *r*^2^ |  |  |
| rs3812036 | 176746010 | 1.48x10^-5^ | 0.963 | 0.897 |  | impute |
| rs11746443 | 176730912 | 1.62x10^-5^ | - | - |  | type |
| rs4074995 | 176729949 | 1.95x10^-5^ | 0.999 | 0.992 |  | type |
| rs35716097 | 176739242 | 1.98x10^-5^ | 0.999 | 0.604 |  | impute |
| rs12654812 | 176726797 | 1.98x10^-5^ | 0.993 | 0.600 |  | type |
| rs4976688 | 176717045 | 3.45x10^-5^ | 0.990 | 0.968 |  | impute |
| rs4075958 | 176717118 | 3.74x10^-5^ | 0.989 | 0.966 |  | type |
| rs10866705 | 176733737 | 7.43x10^-5^ | 1.000 | 0.752 |  | type |
| rs6862195 | 176755118 | 9.41x10^-5^ | 0.933 | 0.642 |  | impute |
| rs4976691 | 176760421 | 9.50x10^-5^ | 0.908 | 0.621 |  | impute |
| Note: Possible role of rs3812036 on SLC34A1. The SNP rs3812036 is absolute linkage of rs12654812, and third strongest association of nephrolithiasis on 5q35.3 using imputation analysis. Top10 SNPs for imputation analysis on 5q35.3 were shown LD for rs11746443. The SNP rs3812036 is located on intron 4. ^a^*P* value obtained from Cochrane-Armitage trend test. | | | | | | |
