# Supplementary material for: A Genome-Wide Association Study of Nephrolithiasis in the Japanese Population Identifies Novel Susceptible Loci at 5q35.3, 7p14.3, and 13q14.1
Source: PLoS Genet. 2012 Mar 1;8(3):e1002541. doi: 10.1371/journal.pgen.1002541 (PMC3291538; doi:10.1371/journal.pgen.1002541)
Supplement: Table S8 — Result of imputation analysis and snp location at 7p14.3 (a) and 13q14.1 (b). (DOCX) [file pgen.1002541.s017.docx]

| **Table S8a Result of imputation analysis at 7p14.3** | | | | | | | | |
| --- | --- | --- | --- | --- | --- | --- | --- | --- |
| SNP | Position | *P^a^* | rs1000597 | |  |  | | status |
|  |  |  | *D* | *r*^2^ |  | gene | location |  |
| rs12669187 | 30882003 | 1.04x10^-6^ | 0.868 | 0.644 |  | *FAM188B* | intronic 15 | type |
| rs12673857 | 30853675 | 1.47x10^-6^ | 0.799 | 0.621 |  | *FAM188B* | intronic9 | impute |
| rs12674155 | 30866068 | 2.47x10^-6^ | 0.805 | 0.626 |  | *FAM188B* | intronic9 | type |
| rs7791980 | 30853025 | 2.95x10^-6^ | 0.793 | 0.616 |  | *FAM188B* | intronic9 | impute |
| rs7792019 | 30853088 | 2.95x10^-6^ | 0.793 | 0.616 |  | *FAM188B* | intronic12 | impute |
| rs12670734 | 30862365 | 3.08x10^-6^ | 0.805 | 0.626 |  | *FAM188B* | intronic9 | impute |
| rs12671536 | 30856854 | 3.16x10^-6^ | 0.764 | 0.569 |  | *FAM188B* | intronic9 | impute |
| rs6959949 | 30853755 | 3.93x10^-6^ | 0.771 | 0.575 |  | *FAM188B* | intronic9 | impute |
| rs1468313 | 30854307 | 4.19x10^-6^ | 0.771 | 0.575 |  | *FAM188B* | intronic9 | type |
| rs12672329 | 30845842 | 8.47x10^-6^ | 0.795 | 0.602 |  | *FAM188B* | intronic7 | impute |
| rs1000597 | 30903703 | 1.06x10^-5^ | - | - |  | *FAM188B-AQP1* | Inter gene | type |
| Note: Possible role of strongly associated on 7p14.3. These SNPs that show the stronger association with nephrolithiasis than rs1000597. Top11 SNPs for imputation analysis on 5q35.3 were shown LD for rs1000597. ^a^*P* value obtained from Cochrane-Armitage trend test. | | | | | | | | |

| **Table S8b Result of imputation analysis at 7p14.3** | | | | | | | | |
| --- | --- | --- | --- | --- | --- | --- | --- | --- |
| SNP | Position | *P^a^* | rs4142110 | |  |  | | status |
|  |  |  | *D* | *r*^2^ |  | gene | location |  |
| rs7981733 | 41588060 | 4.08x10^-7^ | 0.774 | 0.381 |  | *DGKH* | intron1 | type |
| rs912875 | 41575238 | 4.09x10^-7^ | 0.774 | 0.381 |  | *DGKH* | intron1 | impute |
| rs9315891 | 41597079 | 4.12x10^-7^ | 0.774 | 0.38 |  | *DGKH* | intron1 | impute |
| rs1170169 | 41595807 | 1.42x10^-6^ | 0.647 | 0.308 |  | *DGKH* | intron1 | impute |
| rs585206 | 41587853 | 1.75x10^-6^ | 0.647 | 0.308 |  | *DGKH* | Intron1 | impute |
| rs9562369 | 41488075 | 2.78x10^-6^ | 0.507 | 0.044 |  | *KIAA0564-DGKH* | Inter gene | impute |
| rs9562377 | 41583379 | 4.22x10^-6^ | 1 | 0.135 |  | *DGKH* | intron1 | impute |
| rs9566921 | 41585004 | 4.22x10^-6^ | 1 | 0.135 |  | *DGKH* | intron1 | type |
| rs670676 | 41599739 | 5.17x10^-6^ | 0.864 | 0.664 |  | *DGKH* | intron2 | impute |
| rs7984523 | 41576357 | 5.48x10^-6^ | 0.839 | 0.695 |  | *DGKH* | intron1 | impute |
| rs9525568 | 41561251 | 5.78x10^-6^ | 0.717 | 0.341 |  | *DGKH* | intron1 | impute |
| rs1170158 | 41599941 | 6.25x10^-6^ | 0.859 | 0.663 |  | *DGKH* | intron2 | impute |
| rs9566939 | 41680887 | 9.21x10^-6^ | 0.941 | 0.781 |  | *DGKH* | intron17 | type |
| rs347403 | 41687366 | 1.10x10^-5^ | 0.996 | 0.353 |  | *DGKH* | intron20 | impute |
| rs1170155 | 41600711 | 1.15x10^-5^ | 0.889 | 0.528 |  | *DGKH* | intron2 | type |
| rs1170188 | 41576971 | 1.13x10^-5^ | 0.843 | 0.615 |  | *DGKH* | intron1 | impute |
| rs1170187 | 41577835 | 1.13x10^-5^ | 0.843 | 0.615 |  | *DGKH* | intron1 | impute |
| rs1170191 | 41573493 | 1.16x10^-5^ | 0.843 | 0.615 |  | *DGKH* | intron1 | type |
| rs4994103 | 41555148 | 1.17x10^-5^ | 0.918 | 0.119 |  | *DGKH* | intron1 | type |
| rs9566906 | 41540546 | 1.18x10^-5^ | 0.659 | 0.297 |  | *DGKH* | intron1 | type |
| rs9562373 | 41557480 | 1.22x10^-5^ | 0.918 | 0.119 |  | *DGKH* | intron1 | impute |
| rs1900413 | 41558006 | 1.22x10^-5^ | 0.918 | 0.119 |  | *DGKH* | intron1 | impute |
| rs9566935 | 41665185 | 1.29x10^-5^ | 1 | 0.125 |  | *DGKH* | intron14 | type |
| rs17701966 | 41646558 | 1.38x10^-5^ | 1 | 0.125 |  | *DGKH* | intron8 | impute |
| rs1177637 | 41590823 | 1.40x10^-5^ | 0.843 | 0.615 |  | *DGKH* | intron1 | impute |
| rs9566928 | 41606389 | 1.44x10^-5^ | 1 | 0.129 |  | *DGKH* | intron3 | impute |
| rs12876965 | 41610741 | 1.65x10^-5^ | 0.954 | 0.617 |  | *DGKH* | intron3 | impute |
| rs1170102 | 41674117 | 1.67x10^-5^ | 0.986 | 0.619 |  | *DGKH* | intron17 | impute |
| rs1170103 | 41675027 | 1.86x10^-5^ | 0.988 | 0.601 |  | *DGKH* | intron17 | impute |
| rs4598803 | 41660871 | 1.89x10^-5^ | 1 | 0.612 |  | *DGKH* | intron11 | type |
| rs2122247 | 41661140 | 1.90x10^-5^ | 0.988 | 0.601 |  | *DGKH* | intron11 | impute |
| rs10492438 | 41665706 | 1.90x10^-5^ | 0.988 | 0.601 |  | *DGKH* | intron14 | impute |
| rs12584544 | 41666720 | 1.90x10^-5^ | 0.988 | 0.601 |  | *DGKH* | intron14 | impute |
| rs12585267 | 41657971 | 1.93x10^-5^ | 1 | 0.612 |  | *DGKH* | intron11 | impute |
| rs9533007 | 41608235 | 1.94x10^-5^ | 0.992 | 0.602 |  | *DGKH* | intron3 | type |
| rs682573 | 41606655 | 1.97x10^-5^ | 0.954 | 0.617 |  | *DGKH* | intron3 | impute |
| rs12585075 | 41609224 | 1.97x10^-5^ | 0.954 | 0.617 |  | *DGKH* | intron3 | impute |
| rs9566910 | 41549002 | 2.08x10^-5^ | 0.669 | 0.313 |  | *DGKH* | intron1 | impute |
| rs9566924 | 41597907 | 2.19x10^-5^ | 1 | 0.128 |  | *DGKH* | intron1 | type |
| rs9562385 | 41678628 | 2.21x10^-5^ | 0.967 | 0.652 |  | *DGKH* | intron17 | impute |
| rs347413 | 41685040 | 2.61x10^-5^ | 0.985 | 0.601 |  | *DGKH* | intron20 | type |
| rs17598799 | 41670222 | 4.00x10^-5^ | 0.989 | 0.938 |  | *DGKH* | intron15 | impute |
| rs4142110 | 41652522 | 7.15x10^-5^ | - | - |  | *DGKH* | intron10 | type |
| Note: Possible role of strongly associated on 13p14.1. These SNPs that show the stronger association with nephrolithiasis than rs4142110. Top45 SNPs for imputation analysis on 13q14.1 were shown LD for rs4142110. ^a^*P* value obtained from Cochrane-Armitage trend test. | | | | | | | | |
