# Supplementary material for: A Genome-Wide Association Study of Nephrolithiasis in the Japanese Population Identifies Novel Susceptible Loci at 5q35.3, 7p14.3, and 13q14.1
Source: PLoS Genet. 2012 Mar 1;8(3):e1002541. doi: 10.1371/journal.pgen.1002541 (PMC3291538; doi:10.1371/journal.pgen.1002541)
Supplement: Table S9 — Association of rs2835349 with nephrolithiasis in the all stage and combined analysis. (DOCX) [file pgen.1002541.s018.docx]

| **Supplementary Table 9 Association of rs2835349 with nephrolithiasis in the GWAS, replication stage and combined analysis** | | | | | | | | | |
| --- | --- | --- | --- | --- | --- | --- | --- | --- | --- |
| SNP | stage | allele | gene | Case MAF^a^ | Control MAF^a^ | *P*^b^ | OR^c^ | 95%CI^c^ | *P*_het_^e^ |
| rs2835349 | GWAS | a/g | *CLDN14 (21q22.12)* | 0.450 | 0.499 | 6.33x10^-5^ | 1.22 | 1.11-1.35 |  |
|  | Rep1 |  |  |  |  |  |  |  |  |
|  |  |  |  | 0.482 | 0.506 | 3.29x10^-3^ | 1.10 | 1.03-1.18 |  |
|  | Rep2 |  |  | 0.507 | 0.502 | 6.24x10^-1^ | 0.98 | 0.94-1.10 |  |
|  | Combined^d^ |  |  |  |  |  |  |  |  |
|  |  |  |  |  |  | 5.72x10^-4^ | 1.10 | 1.05-1.13 | 0.0014 |
| Note: 5,796 (904 in GWAS and 4,892 in replication) Nephrolithiasis cases and 17,344 (7,471 in GWAS and 9,873 in replication) controls were analyzed. | | | | | | | | | |
| ^a^MAF: minor allele frequency ^b^*P* value Cochrane-Armitage trend test.  ^c^ Odds ratios (OR) and confidence interval (CI) are calculated using the non-susceptible allele as reference.  ^d^Combined: Odds ratio and *P* value for independence test were calculated by Mendel-hauzen and Laird method in the Meta-analysis. ^e^The *P* values of heterogeneities (*P*_het_) across three stages examined by using the Breslow-Day test. | | | | | | | | | |
