# Supplementary material for: A Genome-Wide Association Study of Nephrolithiasis in the Japanese Population Identifies Novel Susceptible Loci at 5q35.3, 7p14.3, and 13q14.1
Source: PLoS Genet. 2012 Mar 1;8(3):e1002541. doi: 10.1371/journal.pgen.1002541 (PMC3291538; doi:10.1371/journal.pgen.1002541)
Supplement: Table S10 — Multiple logistic regression analysis for risk of nephrolithiasis. (DOCX) [file pgen.1002541.s019.docx]

| **Supplementary Table 10 Multiple logistic regression analysis for risk of nephrolithiasis** | | | | | | |
| --- | --- | --- | --- | --- | --- | --- |
| **Covariates** | **Category** | ***P*^a^** | | **OR^b^** | | **95% CI^b^** |
| rs11746443 | TT or TC or CC | 2.36x 10^-10^ | | 1.18 | | 1.12-1.25 |
| rs1000597 | GG or AG or AA | 4.51x 10^-11^ | | 1.21 | | 1.14-1.27 |
| rs4142110 | AA or AG or GG | 6.30x 10^-8^ | | 1.14 | | 1.09-1.19 |
| Age | <60 or ≥60 | <2.0 x 10^-16^ | | 1.91 | | 1.79-2.04 |
| Gender | Female, Male | <2.0 x 10^-16^ | | 2.47 | | 2.30-2.66 |
| BMI | <24 or ≥24 | <2.0 x 10^-16^ | | 2.01 | | 1.88-2.05 |
| Note: 5,230 nephrolithiasis cases and 15,313 controls were used in the logistic regression analysis under additive model. **^a^***P* values were calculated by Wald test. **^b^**Odds ratios (OR) and confidence interval (CI) are calculated using the non-susceptible allele as reference. | | | | | | |
|  | | |  | |  | |
|  | | | | | | |
