# Supplementary material for: A Genome-Wide Association Study of Nephrolithiasis in the Japanese Population Identifies Novel Susceptible Loci at 5q35.3, 7p14.3, and 13q14.1
Source: PLoS Genet. 2012 Mar 1;8(3):e1002541. doi: 10.1371/journal.pgen.1002541 (PMC3291538; doi:10.1371/journal.pgen.1002541)
Supplement: Table S11 — a) SNPs that compose the weighted genetic risk score and weights assigned to each marker. b) wGRS scores and odd ratios of nephrolithiasis by wGRS groups. (DOCX) [file pgen.1002541.s020.docx]

| **Supplementary Table11a　SNPs that compose the wGRS** | | | | | | | |
| --- | --- | --- | --- | --- | --- | --- | --- |
| **Chr^a^** | **SNP** | **Allele** | **Position** | **OR^b^** | **Gene** | **Weight^c^** | **% of total weight** |
| 5 | rs11746443 | A | 176798306 | 1.19 | *RGS14-SLC34A1-PFN3-F12* | 0.1743649 | 34.44% |
| 7 | rs1000597 | G | 30937178 | 1.22 | *INMT-FAM188B-AQP1* | 0.2026357 | 40.02% |
| 13 | rs4142110 | C | 42754522 | 1.14 | *DGKH* | 0.1293185 | 25.54% |
| Note: 5,796 (904 in GWAS, 2,783 in stage2 and 2,109 in stage3) Nephrolithiasis cases and 17,344 (7,471 in GWAS, 5251 in stage2 and 4,622 in stage3) controls were analyzed.  **^a^**Chr: chromosome b**^b^**Odds ratios (OR) are calculated using the non-susceptible allele as reference. **^c^**Weight is the natural log of the odds ratio for each allele | | | | | | | |

| **Supplementary Table11b wGRS and Odd ratios of nephrolithiasis** | | | | |
| --- | --- | --- | --- | --- |
| **Category (wGRS)** | **Case^a^** | **Controls^a^** | **OR^b^** | **95% CI^b^** |
| 1 (-0.127) | 0.051 | 0.077 | 1.00 | reference |
| 2 (0.127-0.309) | 0.438 | 0.490 | 1.34 | 1.17 -1.53 |
| 3 (0.309-0.490) | 0.299 | 0.271 | 1.65 | 1.44 -1.90 |
| 4 (0.490-) | 0.211 | 0.162 | 1.95 | 1.69 -2.25 |
| Note: 5,796 (904 in GWAS, 2,783 in stage2 and 2,109 in stage3) Nephrolithiasis cases and 17,344 (7,471 in GWAS, 5251 in stage2 and 4,622 in stage3) controls were analyzed. We examined 4 categories using wGRS. These categories created from the mean and SD from all samples (Group1< average-1.0SD(0.127), 2=(average-1.0SD(0.127))-(average(0.309)), 3=(average(0.309))-(average+1.0SD(0.490)) and 4>(average+1.0SD(0.490)).  **^a^**Case and Control are frequncy for all categories. **^b^**Odds ratios (OR) and confidence interval (CI) are calculated using category1 as reference. | | | | |
