# Supplementary material for: A Genome-Wide Association Study of Nephrolithiasis in the Japanese Population Identifies Novel Susceptible Loci at 5q35.3, 7p14.3, and 13q14.1
Source: PLoS Genet. 2012 Mar 1;8(3):e1002541. doi: 10.1371/journal.pgen.1002541 (PMC3291538; doi:10.1371/journal.pgen.1002541)
Supplement: Table S12 — Sequences of primers. (DOCX) [file pgen.1002541.s021.docx]

| **Supplementary Table 12 Sequences of Primers** | | | |
| --- | --- | --- | --- |
| Gene | Forward | Reverse | Amplicon size |
| *SLC34A1* | 5’-gtgccacccagactccttac-3’ | 5’-tttctccatggtggcatttc-3’ | 91bp |
| *AQP1* | 5’-ttggacacctcctggctatt-3’ | 5’-ggttgctgaagttgtgtgtga-3’ | 93bp |
| *DGKH* | 5’-ctgcagaggagctcattactagg-3’ | 5’-gccagttcttgctccaaaag-3’ | 70bp |
| *ENST00000434909* | 5’-ggactgagaggctctttgactt-3’ | 5’-ttgttccccaccgggtat-3’ | 190bp |
